# Health financing for universal health coverage in Sub-Saharan Africa: a systematic review

**DOI:** 10.1186/s41256-021-00190-7

**Published:** 2021-03-01

**Authors:** Susan C. Ifeagwu, Justin C. Yang, Rosalind Parkes-Ratanshi, Carol Brayne

**Affiliations:** 1 Department of Public Health and Primary Care, Cambridge Public Health, University of Cambridge, Cambridge, UK; 2 Department of Epidemiology and Applied Clinical Research, Division of Psychiatry, Faculty of Brain Sciences, University College London, London, UK; 3 Infectious Diseases Institute, Makerere University College of Health Sciences, Kampala, Uganda

**Keywords:** Universal health coverage, Health financing, Sub-Saharan Africa, Systematic review

## Abstract

**Background:**

Universal health coverage (UHC) embedded within the United Nations Sustainable Development Goals, is defined by the World Health Organization as all individuals having access to required health services, of sufficient quality, without suffering financial hardship. Effective strategies for financing healthcare are critical in achieving this goal yet remain a challenge in Sub-Saharan Africa (SSA). This systematic review aims to determine reported health financing mechanisms in SSA within the published literature and summarize potential learnings.

**Methods:**

A systematic review was conducted aligned with the Preferred Reporting Items for Systematic Reviews and Meta-Analyses (PRISMA) reporting guidelines. On 19 to 30 July 2019, MEDLINE, EMBASE, Web of Science, Global Health Database, the Cochrane Library, Scopus and JSTOR were searched for literature published from 2005. Studies describing health financing approaches for UHC in SSA were included. Evidence was synthesised in form of a table and thematic analysis.

**Results:**

Of all records, 39 papers were selected for inclusion. Among the included studies, most studies were conducted in Kenya (*n* = 7), followed by SSA as a whole (*n* = 6) and Nigeria (*n* = 5). More than two thirds of the selected studies reported the importance of equitable national health insurance schemes for UHC. The results indicate that a majority of health care revenue in SSA is from direct out-of-pocket payments. Another common financing mechanism was donor funding, which was reported by most of the studies. The average quality score of all studies was 81.6%, indicating a high appraisal score. The interrater reliability Cohen’s kappa score, κ=0.43 (*p* = 0.002), which showed a moderate level of agreement.

**Conclusions:**

Appropriate health financing strategies that safeguard financial risk protection underpin sustainable health services and the attainment of UHC. It is evident from the review that innovative health financing strategies in SSA are needed. Some limitations of this review include potentially skewed interpretations due to publication bias and a higher frequency of publications included from two countries in SSA. Establishing evidence-based and multi-sectoral strategies tailored to country contexts remains imperative.

**Supplementary Information:**

The online version contains supplementary material available at 10.1186/s41256-021-00190-7.

## Background

Access to quality health services, whether preventative or curative, remains a prerequisite in order for a population to attain health and achieve healthy lifestyles [1]. In this context, universal health coverage (UHC), embedded within the 2030 Agenda for Sustainable Development, is defined by the World Health Organization (WHO) as all individuals and communities having access to any health services they need, of sufficient quality to be effective, without suffering financial hardship. With over a hundred million people becoming impoverished annually due to catastrophic health expenditures, particularly in low- and middle-income countries (LMICs), developing solutions is of dire importance [2]. In Sub-Saharan Africa (SSA), 27 out of 48 countries are affected by direct out-of-pocket payments (OOPs) for healthcare services that are greater than 30% (Additional file 1).

A health financing framework was developed by the WHO, highlighting that financing strategies should be integrated within a national health policy and include a service delivery plan [3]. Despite continued global agreement on the need for strengthening national health financing systems to develop sustainable and comprehensive policies, health financing in LMICs and individuals’ access to essential health services depends on OOPs. Such access barriers contribute to high burdens of preventable deaths [4].

In addition, more than approximately 800 million people spend at least 10% of their income on health care through OOPs, which pushes millions of individuals further into poverty each year [5]. Strengthening of domestic financing is crucial to avoid OOPs and it was emphasised that countries must increase their allocated spending on primary health care by at least 1% of their gross domestic product (GDP) if health targets of the 2030 Agenda for Sustainable Development are to be met [6].

With the unprecedented emergence of the coronavirus disease 2019 (COVID-19) pandemic, UHC is more important than ever. Health care access and quality remain a challenge worldwide and efforts to improve these issues through UHC are pivotal. Effective strategies for financing healthcare are critical yet remain a challenge in most of SSA. In this respect, the aim of the systematic review is to determine the reported health financing mechanisms in SSA within published literature and ascertain potential learnings and successful strategies for countries in the region.

## Methods

### Protocol and registration

A systematic literature review was conducted in line with the Preferred Reporting Items for Systematic Reviews and Meta-Analyses (PRISMA) reporting guidelines. The review protocol was registered with the International Prospective Register of Systematic Reviews (PROSPERO) on 14 August 2019 (registration number CRD42019142895, Additional file 2).

### Search strategy

On 19 to 30 July 2019, MEDLINE, EMBASE, Web of Science, Global Health Database, the Cochrane Library, Scopus and JSTOR were searched for literature published from 2005 (Additional file 3). The period focussed on, but was not limited to, research from 2005 till 30 July 2019, the final date of the search, as that is when global commitment by the WHO Member States was further refined for the transition to UHC. The search included terms related to UHC and all countries in SSA in order not to limit findings. Different keywords and Medical Subject Headings (MeSH) terms were used to conduct the search throughout the various databases. In order to capture the different terms for UHC, Boolean operators, such as “OR”, were used.

### Study selection and eligibility criteria

All studies, both qualitative and quantitative, which described health financing approaches and strategies for UHC in SSA were eligible for inclusion. The inclusion criteria involved literature published in 2005 to 30 July 2019, English or French studies, studies concerning health financing strategies and policies for healthcare services in the context of UHC in LMICs of SSA and vulnerable populations (Table 1). Examples included topics related to funding, financing systems, financial protection, OOPs, pooling of funds, revenue raising, benefit package designs and purchasing of services, among others. Studies that did not mention UHC were excluded. For selected papers, reference lists were searched through the snowballing procedure for further studies.

**Table 1:** PICo (Population, Intervention, Context) framework to structure the review question

*Framework Item*		Details
P	***P****atient,* ***P****opulation, or* ***P****roblem*	Sub-Saharan African population
I	***I****ntervention or Exposure*	Health financing approaches for universal health coverage (UHC)
Co	***C****ontext*	Sub-Saharan Africa

### Data extraction and items

Records in the form of titles and abstracts retrieved from the search strategy were collected by two independent reviewers (JY and SI) and screened independently according to the inclusion and exclusion criteria using reference management systems. Once the status of inclusion or exclusion of the study had been assessed, the selected studies for review were re-assessed to confirm their suitability. Next, full text studies were assessed for their suitability. A second reviewer confirmed the selection based on the criteria. The data extraction method followed the guidance of the Cochrane Collaboration Qualitative Methods Group and established a suitable, standardised data extraction form. For selected studies, the main data extracted to a Microsoft Excel spreadsheet included the citation information, year of publication, setting, population, study objective, methods, design, health financing approaches, services covered, funding agencies, limitations, conclusions and recommendations.

### Method of synthesis

The strategy for data synthesis involved a narrative description and thematic synthesis of the selected studies in the form of tables and text. Evidence was synthesised based on the setting, population, health financing approaches, as well as limitations, conclusions and recommendations for further research. Given the qualitative nature of the studies, the planned analytical approach included the categorisation of studies and identification of potential recurring themes within the narrative synthesis. The descriptive themes were developed based on the findings and aimed to answer the questions specific to the review and provide a thematic structure for the commentaries, learnings and reflections of selected studies.

### Quality appraisal and risk of bias

The quality assessment and critical appraisal of the selected studies, which included assessing the risk of bias, involved the use of the Critical Appraisal Skills Programme (CASP) Qualitative Checklist and Joanna Briggs Institute (JBI) Appraisal Tools. Checklists for qualitative, quantitative, mixed methods and systematic reviews were completed for each of the individual studies included and a final score was provided in form of a percentage score (1 to 100). The assessment was conducted independently by both assessors. Finally, the outcomes were combined into a single quality score for the individual studies, by combining both final scores and dividing by two. To facilitate the comparison of the studies, as feasible, the scoring was grouped into low (a score < 60), medium (60–80) and high (> 80). The degree of agreement (interrater reliability) was measured using Cohen’s kappa (κ).

### Summary results of the critical appraisal

Following the combination of the two independent appraisals by both researchers, results indicated that a majority of the studies (*n* = 22) obtained a high score (> 80%). Fifteen studies obtained a medium score (60–80%), while two studies received a low rating (< 60%). On average, the quality score of all studies combined was 81.6%, which indicates an overall high appraisal score across the selected studies (Additional file 4). The kappa score, κ=0.43 (*p* = 0.002), showed a moderate level of agreement. Notwithstanding any of the lower scoring studies, all studies were included in the final thematic analysis due to the limited number marked for inclusion.

### Data analysis

The data extracted from the selected studies were summarised and organised into relevant topics based on the thematic analysis. Similarities within concepts, challenges, conclusions and recommendations were identified and grouped within the narrative summary accordingly.

## Results

### Main summary of study selection and results

**Fig. 1:**
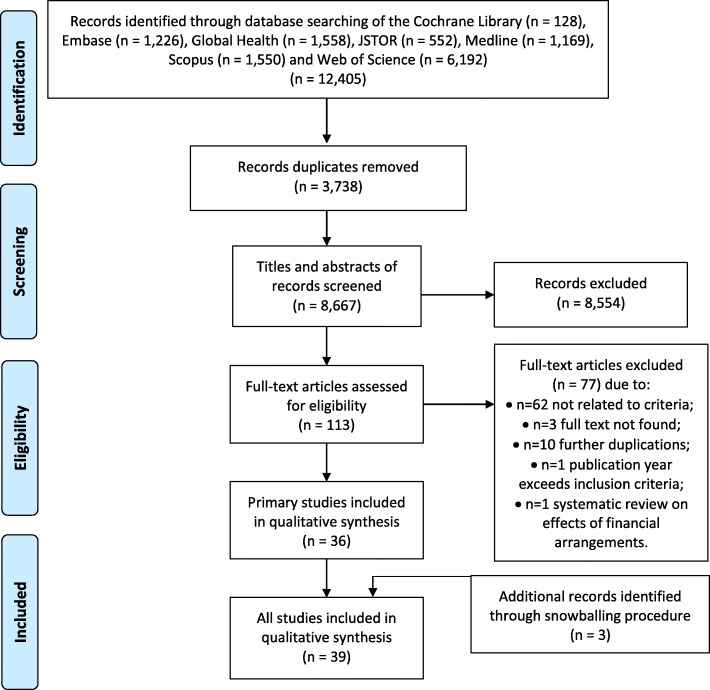
PRISMA flow chart to show the study selection process. Source: flowchart adapted from the available template provided by PRISMA: http://prisma-statement.org/

Of the screened records throughout the review, 39 papers were selected for inclusion, which included three additional records via the snowballing procedure (Fig. 1).

**Fig. 2:**
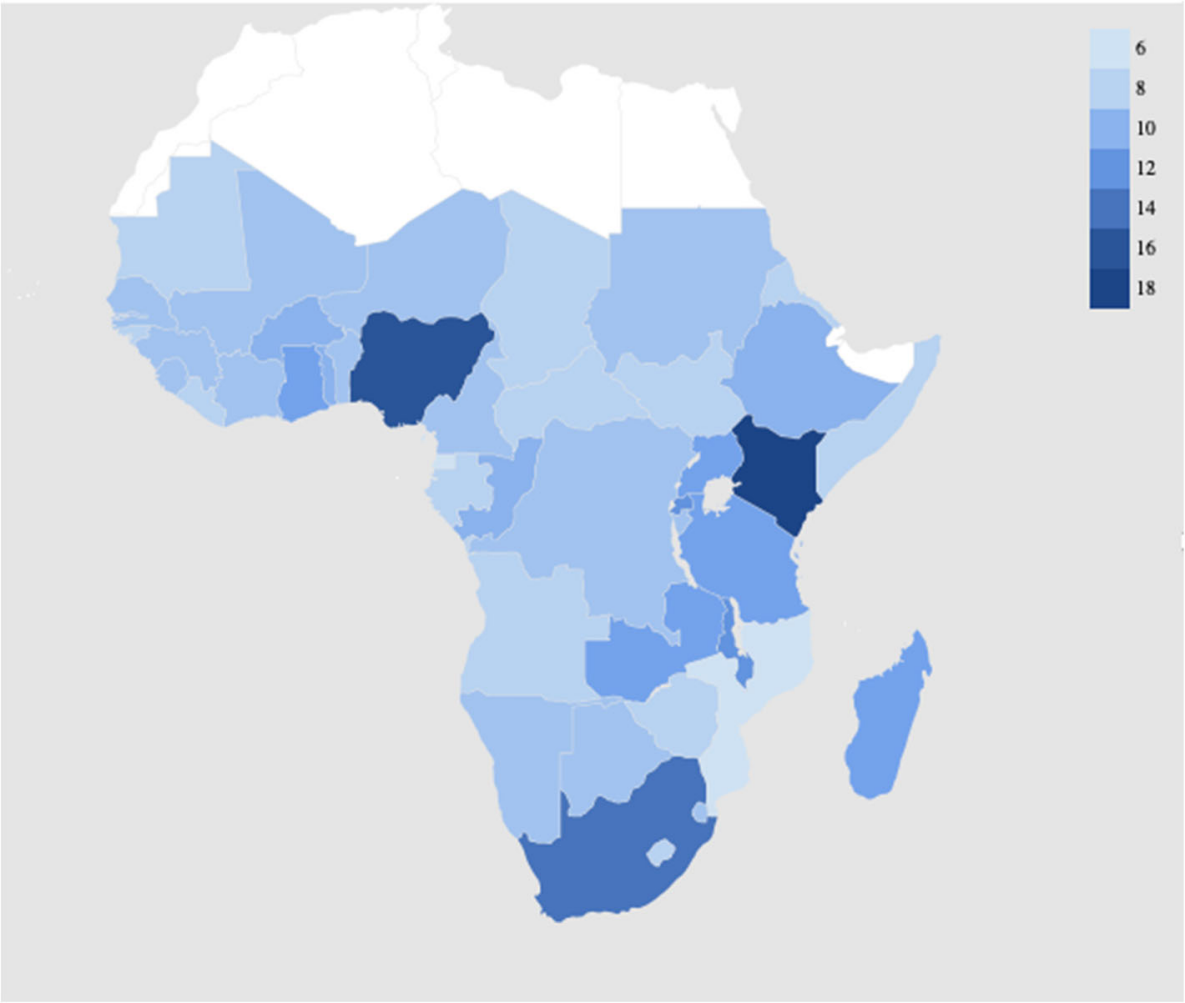
Choropleth map showing the frequency of countries reported in the studies (*n* = 39). Note: Countries filled in white are not included in SSA

The frequency of countries reported in the 39 studies varied, with a majority of studies from Kenya (*n* = 7), followed by SSA (*n* = 6) as a region and Nigeria (*n* = 5) (Fig. 2). Recurring themes that emerged evolved around national or social health insurance, community-based health insurance, tax-based financing, donor funding, and other forms of insurance such as private, voluntary and micro health insurance. For the analysis, findings were categorised using the abovementioned themes. A comprehensive table of the extracted data is displayed in the Additional file 4.

**Fig. 3:**
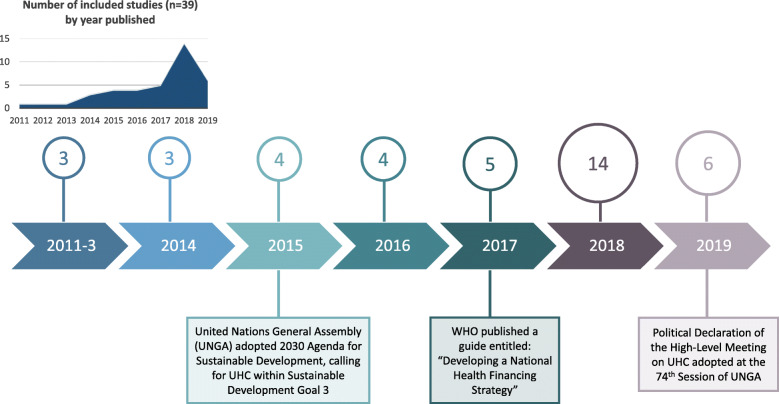
Timeline of UHC and health financing milestones and the distribution of studies (*n* = 39) by year

**Fig. 4:**
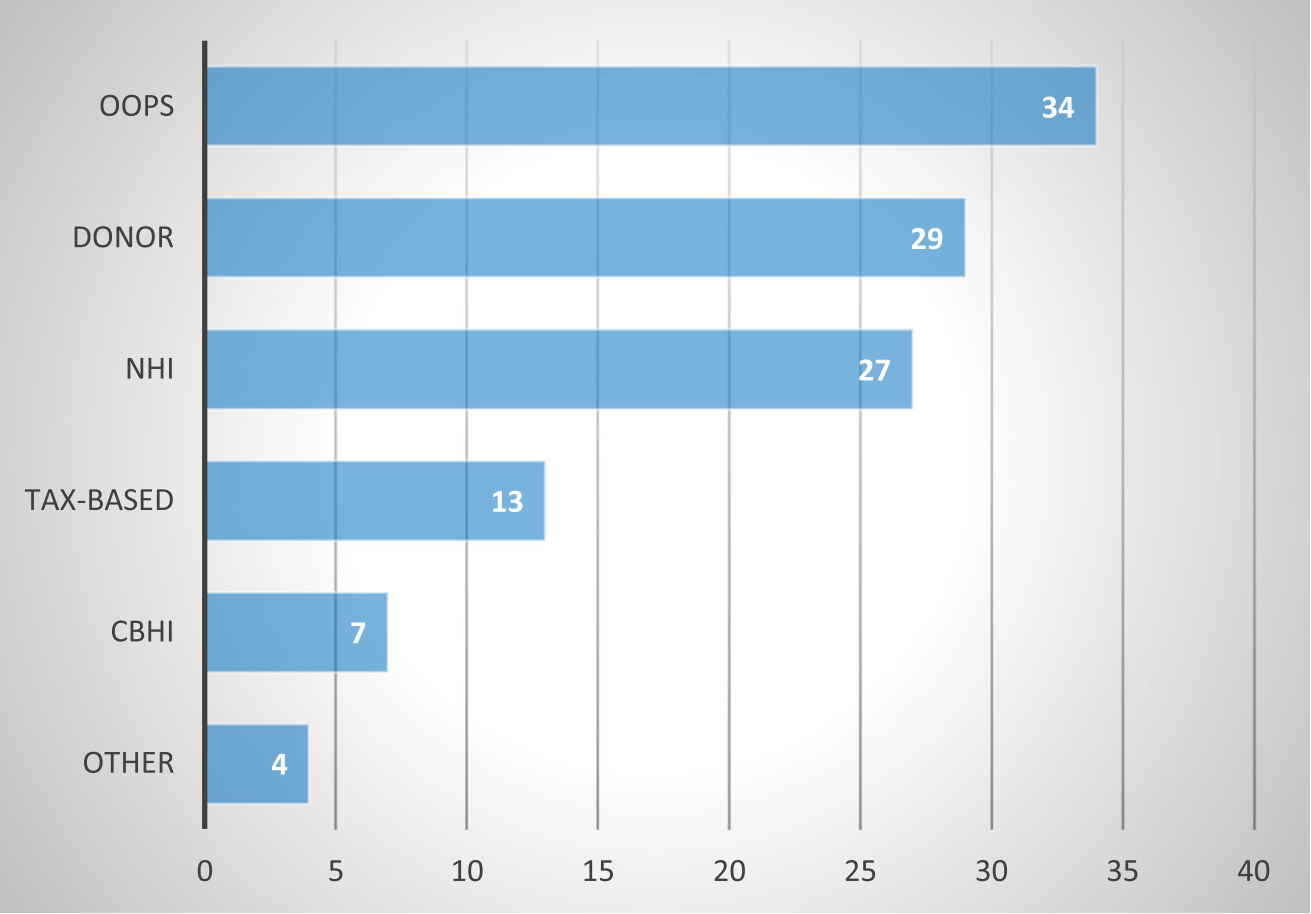
Health financing mechanisms reported among the selected studies (*n* = 39). *Note: ‘Donor’ = external donor funding; ‘OOPs’ = Out-of-pocket payments; ‘NHI’ = national health insurance; ‘Tax-based’ = tax-based financing; ‘CBHI’ = Community-based health insurance; and ‘Other’ = voluntary/micro health insurance

Most of the included studies were published more recently, suggesting that research on health financing for UHC in SSA has increased over time, possibly due to important UHC milestones, more visibility or funding for related projects; this is visually represented in a timeline (Fig. 3). The articles reviewed were focused on the issue of health care revenue in SSA from direct OOPs (*n* = 34) (Fig. 4 and Additional file 5) [7–24]. Another common health financing mechanism throughout the region was external, donor funding, which was reported by most of the studies included (*n* = 29) [8, 9, 11, 13–17, 20–22, 24–30]. Overall, many countries are starting to develop national health insurance schemes, while others already have certain structures in place despite low population coverage of these schemes [31].

#### National or social health insurance

According to the selected studies, a number of countries are either in the process of fully implementing national health insurance (NHI) as a sustainable health financing mechanism or already have such structures in place. A single, compulsory NHI provides the most equitable option, as voluntary health insurances can lead to further inequities and disparities among populations [18, 32]. Social health insurance (SHI), a compulsory system that deducts contribution payments directly from employee payroll taxes is another health financing mechanism. However, in LMICs in SSA, where the formal sector is relatively small and a majority of the population are in the informal sector, this approach is less suitable and sustainable [31].

#### Community-based health insurance (CBHI)

While a minor number of individuals enrolled in CBHI schemes in SSA, such as the Rwandan ‘Mutuelles de Santé’, experience improvements in access to services, it is inequitable across populations and the retention of its members is a challenge, undermining their sustainability and usefulness in supporting progress towards UHC [33]. Other examples have shown that despite CBHI schemes being developed in Kenya through the support of external funding, they consecutively ceased following the discontinuation of funding by donor funds, suggesting the importance of sustained donor support for such schemes [34].

#### Tax-based financing

In tax-based health systems, whole populations have access to health services, irrespective of their socio-economic, as finances for health care are collected by the government from tax revenues [35]. In order to accelerate progress to UHC in Nigeria, a recent study proposed that national policy makers should consider a tax-based, non-contributory, universal health financing system as the principal mechanism [25]. Other examples, in Kenya, suggested the utilisation of tax funds to provide subsidies for the poor and the simultaneous development of a framework to help identify the poor and to whom these subsidies would be dispersed to [36].

#### External funding

As a region, SSA relies on donor funding and assistance [37, 38], which suggests a level of dependence on external actors and undermines sustainability efforts. Notably, the Rwandan experience has shown that without donor support for the CBHI scheme, sustainable health insurance coverage, particularly subsidising poor populations, would have been impossible [31]. Effective use of external funding relies on good governance and leadership. This type of financing mechanism, albeit helpful, may lead to further challenges as funds allocated for vertical programmes, to target specific diseases, may distort actual health care priorities in countries, result in inefficiencies in the health system and displace domestic resources [32].

#### Other forms of health insurance

Other types of health insurance, such as voluntary or private health insurance (PHI) and micro health insurance are discussed among a limited number of studies (*n* = 4). Both are more regressive, less equitable types of insurance as they are predominantly affordable by the richer segment of the population, leading to further inequities and are ineffective strategies for UHC financing [39, 40]. However, in conjunction with other mechanisms such issues may be averted. For instance, in South Africa, the collective effect of general taxes and PHI outweighed the regressive nature of OOPs and thus made health financing progressive overall [39].

#### Innovative financing approaches

Some of the innovative health financing approaches mentioned included increasing government health expenditure, developing tax-based systems, improving tax compliance and revenue collection efficiency and implementing so-called “sin taxes” on alcohol, tobacco and air travel earmarked for the health sectors and as financing mechanisms for UHC [33, 41]. An alternative solution to increase health financing in Ethiopia was to incorporate a “sin tax” on “*khat”*, a stimulant substance that is traditionally used in the country [42, 43]. Other potential approaches for raising funding for health include levies on mobile phone call tariffs, raising diaspora bonds in which nationals working abroad provide additional financial support, and the taxation of profitable sectors, such as the banking or petroleum industry [33].

#### Learnings and successful strategies

Lessons learned across all studies included the importance of enhancing public-private partnerships (PPPs) by leveraging the potential of the private sector to complement public sector efforts and developing innovative domestic financing by improved efficiency in tax collection to support the generation of these finances [8, 9, 17, 19–24, 26, 28, 29, 31–33, 37, 38, 41, 42, 44–47]. Resilience and the role of government in increasing financial investment were key factors that led to the success of the Rwandan case [17, 31]. Cultural solidarity and resilience were important for the success of the CBHI scheme in Uganda [10]. Ghana’s SHI scheme was another successfully implemented strategy [27]. Besides increased health spending, additional pooled resources for UHC, strengthening capacity, and socio-political factors and policy levers need to be established [13].

## Discussion

UHC is, arguably, one of the most important aspects of equitable and fair access to health care services relevant to the needs of individuals. Access to quality health services without individuals suffering financial hardship remains a challenge in LMICs. In this regard, the systematic review sought to determine the reported health financing mechanisms in SSA and ascertain potential learnings for countries in the region. Solutions are not a ‘one size fits all’, generalisable model, but require tailored, country-specific approaches that address the unique needs of countries. The small number of studies included suggest that while there seems to be a scarcity of published literature on this subject area, publications are increasing over time possibly due to heightened global impetus. The findings also reveal geographic gaps in the literature, specifically francophone countries in SSA, which could be due to more limited reporting or publishing in these areas.

There is a need for the reliance on OOPs to be reduced to provide financial protection and improve affordability and access to health services, as discussed in 34 of the 39 included studies. As a repercussion of reliance on regressive forms of payments, millions of people either do not seek treatment for their health needs, exacerbating the problem, or, alternatively, if they do, it may result in financial hardship to them [48]. Solely in circumstances where OOPs are less than 15–20% of a country’s total health expenditure, only in approximately one fourth of countries in SSA (*n* = 11), does the incidence of financial catastrophe fall to negligible levels [48].

Several challenges persist, which are commonly noted across the included studies. Ensuring that quality health services reach some of the most vulnerable communities remains a challenge throughout the region. Basic amenities for health facilities, such as water, electricity, sanitation, are lacking in rural areas and require investment. A considerable challenge is the dependency on donor funding, which was highlighted in 29 studies, and developing effective strategies to strengthen domestic financing mechanisms. As indicated by the Rwandan experience, sustainable health insurance coverage required significant donor support. Thus, efforts made by donor countries that advocate for UHC ought to be prepared to provide substantial amounts of funding for prolonged periods to maintain positive health outcomes [31]. Communicating return on investments to donors and considering priority investment areas while sustaining financing commitments to support countries to provide basic healthcare remain critical.

Concerted efforts at various levels, between local governments, donors and private sectors, will be necessary to provide resources targeted for vulnerable population groups [44]. More than half of the included studies (*n* = 23) emphasised the value of PPPs in health financing reforms, requiring coordinated efforts to address quality, efficiency and financing issues in health service delivery [45]. Over two thirds (69%) of the selected studies reported the importance of equitable NHI schemes for UHC alongside additional financing mechanisms. A disconnect often exists between high-level policy makers, practitioners, who have the operational expertise, and academic researchers, who produce the evidence base, culminating in research-to-policy or policy-to-implementation gaps [47]. A stronger platform for dialogue amongst these groups is needed while enhancing the engagement of the public and academia in the public discourse and policy design stage [34]. In addition, there seems to be a lack of reporting advocacy efforts for UHC in the region, suggesting a research gap in this area.

### Recommendations for further research

Further research should focus on UHC within all SSA countries to strengthen evidence-based policy development. Particularly, addressing some of the identified complex research gaps, such as advocacy, performance monitoring, impact evaluation, return on investment or dynamics of stakeholders’ collaboration. Few studies reported information related to the three dimensions of the UHC cube, thus particularly research on the extent of financial risk protection and service coverage of the informal sector is needed.

### Limitations

A number of limitations arose throughout the course of the systematic review. Data from national policy documents were not included as this was beyond the scope of the research question as a systematic review of peer reviewed literature. Therefore, reporting and publication bias may have restricted potentially relevant studies and encouraged selective outcome reporting. Policy documents could provide imperative data and information not presented in the literature. To overcome some of these related limitations, further analyses will be supported by information from these sources to establish more complete interpretations. Finally, as shown in Fig. 2, results obtained could have been skewed given the frequency of certain countries reported in the region. Of the final selection of studies, some referred to the entire region while others were specific to one country, thus impacting the reflections and conclusions. Kenyan and Nigerian studies made up almost one third of the total number of studies.

## Conclusions

To reiterate, the thematic analysis posits that strategies for health financing of UHC ought to be attuned to contextual settings. There is a need for evidence-based, coordinated and multi-sectoral strategies tailored to country contexts to provide sustainable solutions for this complex issue. Particularly, in view of the unprecedented emergence of the COVID-19 pandemic, appropriate health financing mechanisms to support UHC and sustainable health services are vital. Further research could explore COVID-19 in context of global health security, resilience and capacity-building for UHC.

## Supplementary Information

**Additional file 1.**

**Additional file 2.**

**Additional file 3.**

**Additional file 4.**

**Additional file 5.**

## Data Availability

The data generated from this review is provided in the supplementary information files.

## Abbreviations

CASP: Critical Appraisal Skills Programme
CBHI: Community-based health insurance
CHE: Current Health Expenditure
COVID-19: Coronavirus disease 2019
GDP: Gross Domestic Product
JBI: Joanna Briggs Institute
LMICs: Low- and middle-income countries
MeSH: Medical Subject Headings
NHI: National health insurance
OOPs: Out-of-pocket payments
PHI: Private health insurance
PRISMA: Preferred Reporting Items for Systematic Reviews and Meta-Analyses
PPP: Public-Private Partnerships
PROSPERO: International Prospective Register of Systematic Reviews
SSA: Sub-Saharan Africa
SHI: Social health insurance
UHC: Universal health coverage
UNGA: United Nations General Assembly
WHO: World Health Organization
