# Supplementary material for: Health financing for universal health coverage in Sub-Saharan Africa: a systematic review
Source: Glob Health Res Policy. 2021 Mar 1;6:8. doi: 10.1186/s41256-021-00190-7 (PMC7916997; doi:10.1186/s41256-021-00190-7)
Supplement: Supplementary file 1 — Additional file 1. [file 41256_2021_190_MOESM1_ESM.docx]

Table 1. Background statistics and health financing indicators from all countries in Sub-Saharan Africa (n=48).

| Country | Population size (thousands), most recent value in 2019^(a)^ | Gross Domestic Product (GDP) per capita (current US$), 2019^(a)^ | Current health expenditure (CHE) of government (as % of GDP), 2017^(a)^ | Out-of-pocket payment (OOP) expenditure (as % of CHE, 2017^(a)^ | Social Health Insurance (SHI) as % of CHE, 2017, as available^(b)^ | Domestic General Government Health Expenditure as % of CHE, 2017, as available^(b)^ | Coverage of social insurance programs as a % of the population (most recent year in parentheses) ^(a)^ | Universal health coverage (UHC) service coverage index, 2017^(b)^ |
| --- | --- | --- | --- | --- | --- | --- | --- | --- |
| Angola | 31,825.29 | 2,973.6 | 2.79 | 34.12 | 0 | 46 | N/A | 40 |
| Benin | 11,801.15 | 1,219.4 | 3.72 | 44.98 | 2 | 30 | 7 (2003) | 40 |
| Botswana | 2,303.70 | 7,961.3 | 6.13 | 2.99 | 0 | 76 | 4 (2015) | 61 |
| Burkina Faso | 20,321.38 | 774.8 | 6.92 | 31.68 | 0 | 43 | 2 (2014) | 40 |
| Burundi | 11,530.58 | 261.2 | 7.52 | 25.46 | 1 | 25 | N/A | 42 |
| Cabo Verde, Republic of | 549.93 | 3,603.8 | 5.17 | 28.90 | 16 | 60 | 6 (2007) | 69 |
| Cameroon | 25,876.38 | 1,497.9 | 4.67 | 70.96 | 0 | 13 | 3 (2014) | 46 |
| Central African Republic | 4,745.19 | 467.9 | 5.82 | 31.19 | 0 | 13 | N/A | 33 |
| Chad | 15,946.88 | 709.5 | 4.49 | 58.02 | 0 | 16 | 2 (2011) | 28 |
| Comoros | 850.89 | 1,393.5 | 7.38 | 74.65 | 3 | 13 | 2 (2004) | 52 |
| Congo, Democratic Republic of | 86,790.57 | 545.2 | 3.98 | 40.12 | 1 | 10 | 2 (2012) | 41 |
| Congo, Republic | 5,380.51 | 2,011.1 | 2.93 | 48.40 | 0 | 41 | 9 (2005) | 39 |
| Côte d'Ivoire | 25,716.54 | 2,286.2 | 4.45 | 39.36 | 2 | 28 | 8 (2015) | 47 |
| Equatorial Guinea | 1,355.99 | 8,131.9 | 3.11 | 76.79 | 1 | 19 | N/A | 45 |
| Eritrea | 3,213.97** | 642.5 | 2.87 | 59.12 | 0 | 27 | N/A | 38 |
| Eswatini | 1,148.13 | 3,837.0 | 6.93 | 10.50 | 0 | 51 | 6 (2016) | 63 |
| Ethiopia | 112,078.73 | 857.5 | 3.50 | 34.41 | 0 | 25 | 2 (2015) | 39 |
| Gabon | 2,172.58 | 7,667.4 | 2.78 | 25.16 | 24 | 63 | 13 (2005) | 49 |
| Gambia, The | 2,347.71 | 751.3 | 3.28 | 22.03 | 0 | 23 | 2 (2010) | 44 |
| Ghana | 30,417.86 | 2,202.1 | 3.26 | 40.29 | 10 | 33 | 48 (2016) | 47 |
| Guinea | 12,771.25 | 1,064.1 | 4.12 | 56.68 | 2 | 17 | 2 (2012) | 37 |
| Guinea-Bissau | 1,920.92 | 697.8 | 7.24 | 72.06 | 1 | 8 | N/A | 40 |
| Kenya | 52,573.97 | 1,816.5 | 4.80 | 24.04 | 8 | 43 | 1 (2015) | 55 |
| Lesotho | 2,125.27 | 1,157.5 | 8.76 | 16.64 | 0 | 63 | 1 (2017) | 48 |
| Liberia | 4,937.37 | 621.9 | 8.16 | 45.51 | 0 | 17 | 0 (2016) | 39 |
| Madagascar | 26,969.31 | 522.2 | 5.50 | 24.69 | 0 | 47 | N/A | 28 |
| Malawi | 18,628.75 | 411.6 | 9.65 | 10.59 | 0 | 31 | 1 (2016) | 46 |
| Mali | 19,658.03 | 890.7 | 3.79 | 35.11 | 10 | 35 | 1 (2009) | 38 |
| Mauritania | 4,525.70 | 1,677.9 | 4.40 | 49.55 | 10 | 39 | 9 (2008) | 41 |
| Mauritius | 1,265.71 | 11,203.5 | 5.72 | 48.87 | 0 | 43 | 19 (2012) | 63 |
| Mozambique | 30,366.04 | 491.8 | 4.94 | 7.40 | 2 | 30 | 4 (2014) | 46 |
| Namibia | 2,494.53 | 4,957.5 | 8.55 | 7.73 | 0 | 46 | 4 (2015) | 62 |
| Niger | 23,310.72 | 554.6 | 7.74 | 47.83 | 1 | 33 | 1 (2014) | 37 |
| Nigeria | 200,963.60 | 2,229.9 | 3.76 | 77.22 | 1 | 14 | 3 (2015) | 42 |
| Rwanda | 12,626.95 | 801.7 | 6.57 | 6.25 | 12 | 34 | 11 (2013) | 57 |
| São Tomé and Principe | 215.06 | 1,994.9 | 6.23 | 13.78 | 1 | 46 | N/A | 55 |
| Senegal | 16,296.36 | 1,446.8 | 4.13 | 52.40 | 4 | 21 | 8 (2011) | 45 |
| Seychelles | 97.63 | 17,401.7 | 5.01 | 24.60 | 0 | 73 | N/A | 71 |
| Sierra Leone | 7,813.22 | 504.5 | 13.42 | 50.41 | 0 | 14 | N/A | 39 |
| Somalia | 15,442.91 | 126.9 | N/A | N/A | N/A | N/A | N/A | 25 |
| South Africa | 58,558.27 | 6,001.4 | 8.11 | 7.77 | 0 | 54 | 3 (2014) | 69 |
| South Sudan | 11,062.11 | 1,119.7 | 9.76 | 19.19 | 0 | 8 | N/A | 31 |
| Sudan | 42,813.24 | 441.5 | 6.34 | 72.48 | 11 | 18 | N/A | 44 |
| Tanzania, United Republic of | 58,005.46 | 1,122.1 | 3.65 | 24.10 | 8 | 43 | 1 (2014) | 43 |
| Togo | 8,082.37 | 675.5 | 6.20 | 58.42 | 3 | 18 | 3 (2011) | 43 |
| Uganda | 44,269.59 | 776.8 | 6.19 | 39.07 | 0 | 16 | 1 (2012) | 45 |
| Zambia | 17,861.03 | 1,291.3 | 4.47 | 11.85 | 0 | 39 | 1 (2015) | 53 |
| Zimbabwe | 14,645.47 | 1,464.0 | 6.64 | 20.62 | 0 | 52 | 5 (2019) | 54 |

*Sources*: (a) Population size, Gross Domestic Product (GDP) per capita, Current health expenditure (CHE), Out-of-pocket payment (OOP) expenditure and coverage of social insurance programs data derived from the World Bank Open Data: <https://data.worldbank.org/>. Date Accessed: 7 October 2020. (b) Social Health Insurance (SHI), domestic general government health expenditure and UHC service coverage index data made available from the WHO Global Health Expenditure Database: <https://apps.who.int/nha/database>. Date Accessed: 7 October 2020.

*Notes:* N/A = data not available. The Sub-Saharan African region, n=48 countries, is defined as reported by the World Bank and World Health Organization; **Data from most recent value in 2011.
