# Supplementary figures and images for: Health financing for universal health coverage in Sub-Saharan Africa: a systematic review
Source: Glob Health Res Policy. 2021 Mar 1;6:8. doi: 10.1186/s41256-021-00190-7 (PMC7916997; doi:10.1186/s41256-021-00190-7)

## Additional file 2. A copy of the online PROSPERO registration


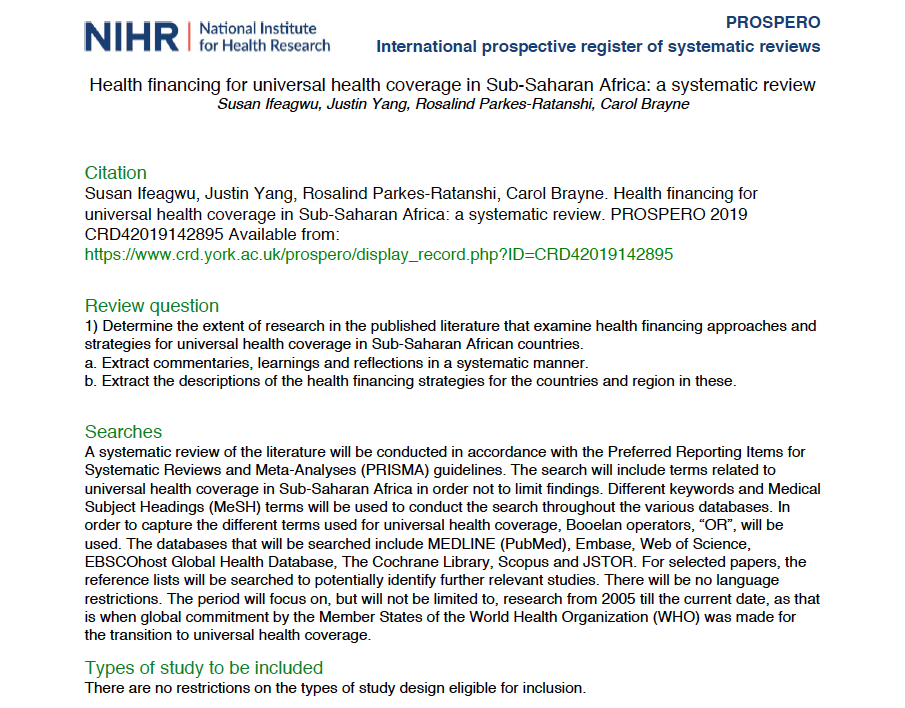

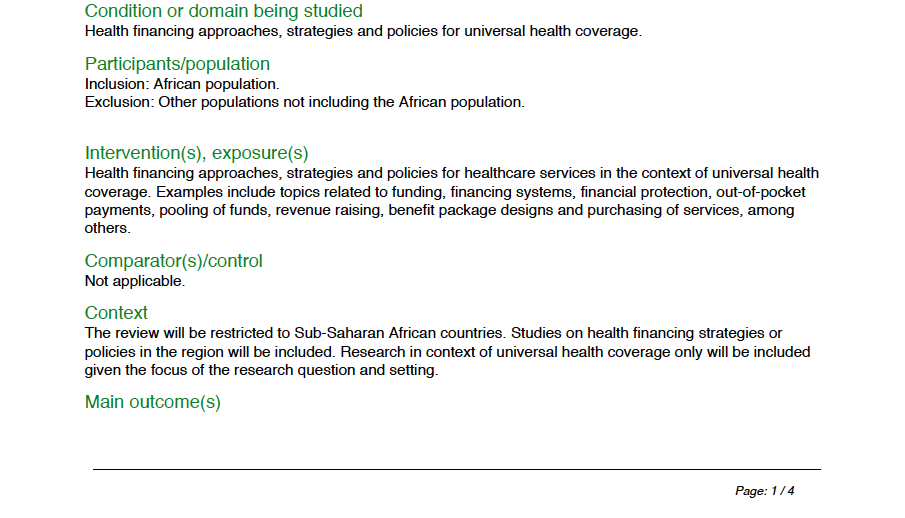


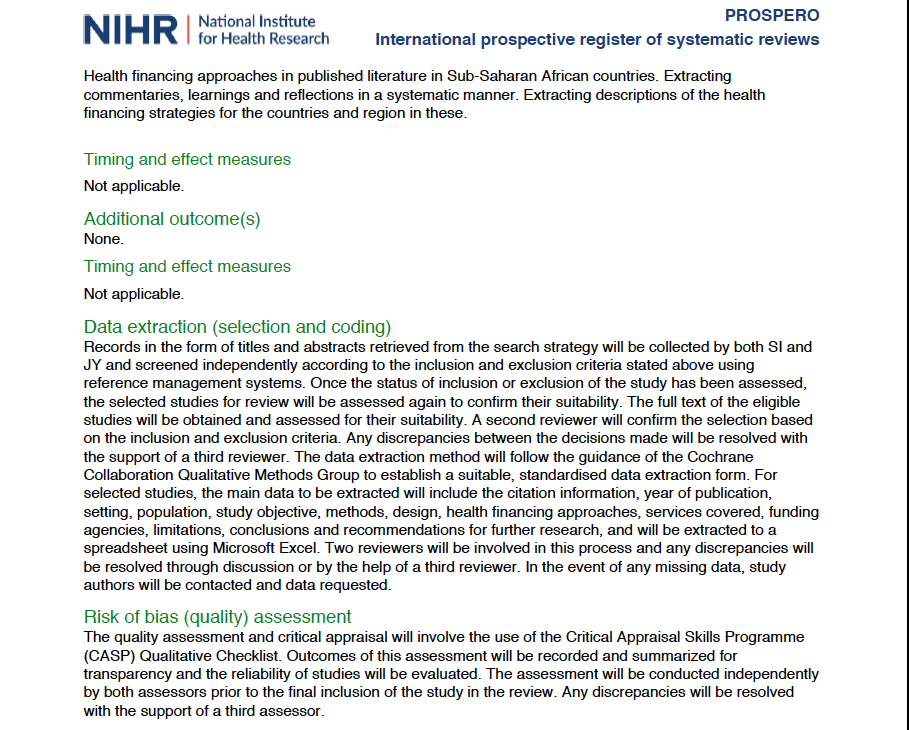

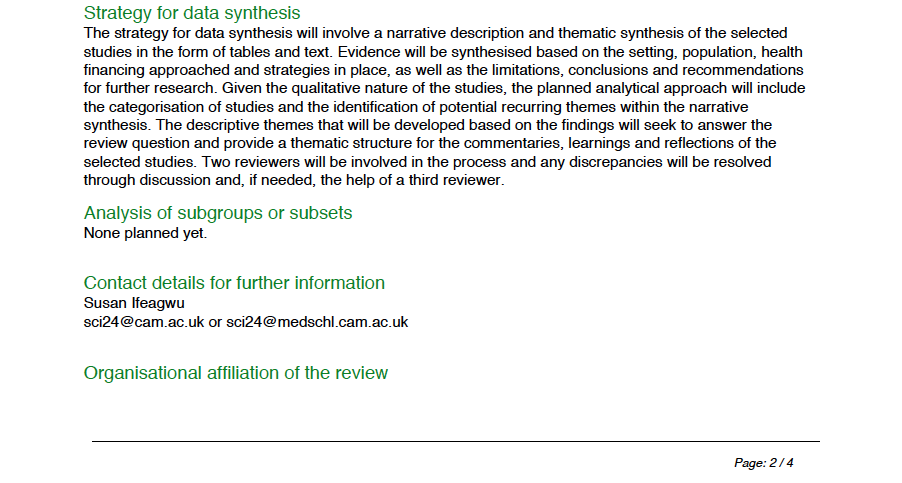


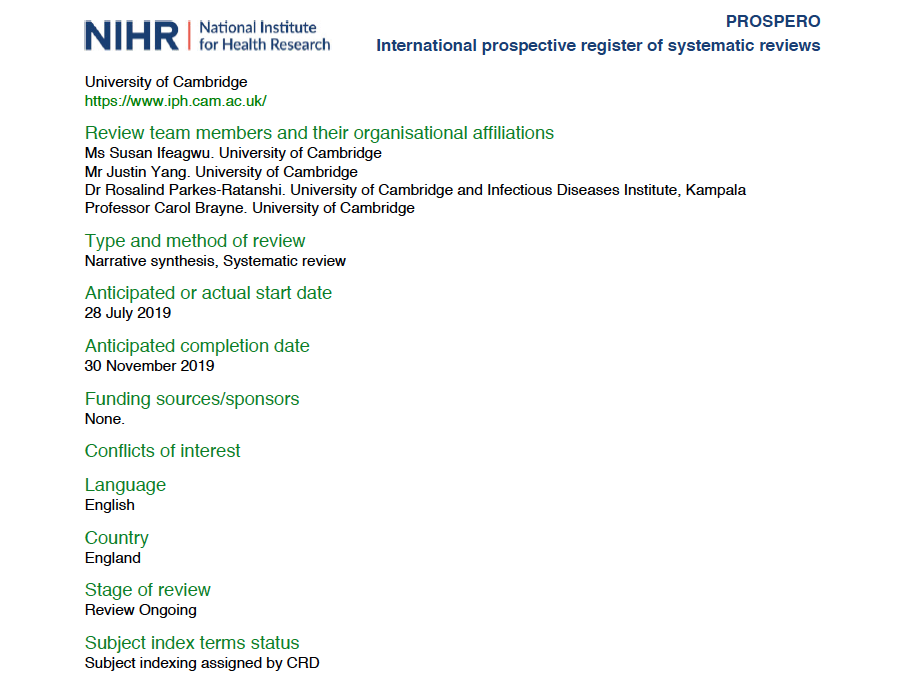

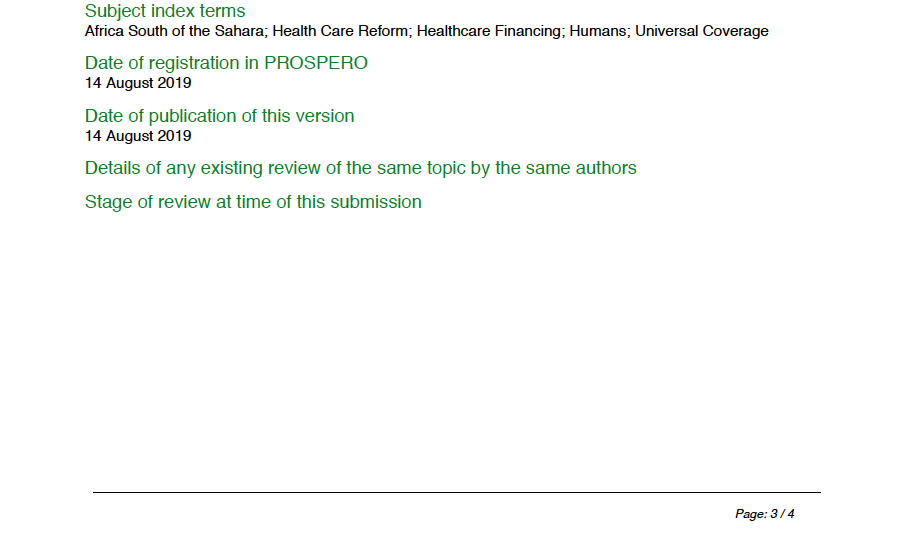


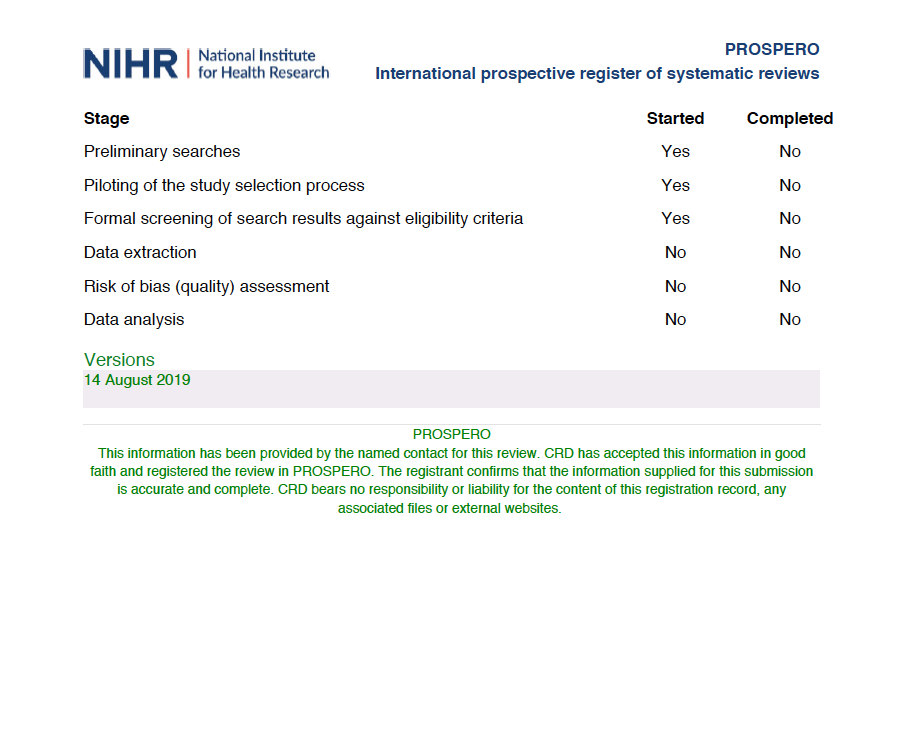

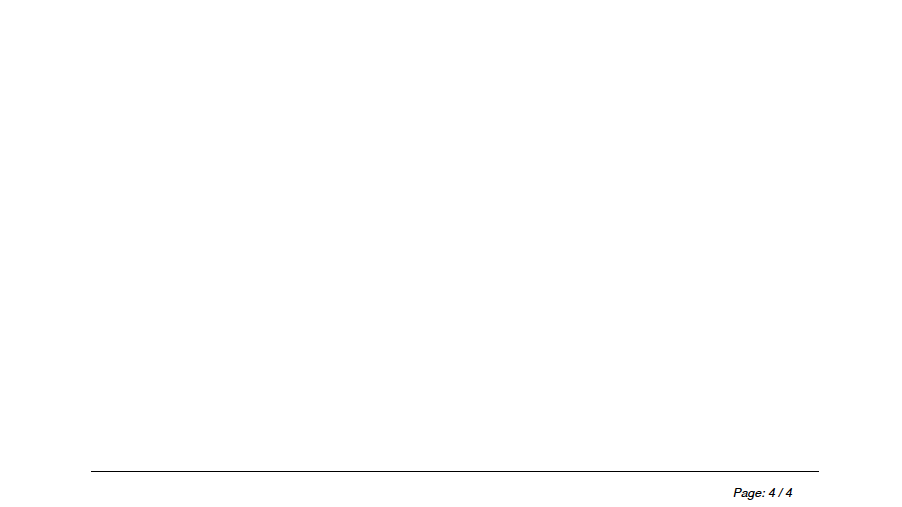

Supplement: Supplementary file 2 — Additional file 2. [file 41256_2021_190_MOESM2_ESM.docx]
