# Supplementary material for: Health financing for universal health coverage in Sub-Saharan Africa: a systematic review
Source: Glob Health Res Policy. 2021 Mar 1;6:8. doi: 10.1186/s41256-021-00190-7 (PMC7916997; doi:10.1186/s41256-021-00190-7)
Supplement: Supplementary file 3 — Additional file 3. [file 41256_2021_190_MOESM3_ESM.docx]

## Additional file 3. Search strategy and results obtained from systematic database search

**Brief Summary**

| **Details:** | Final search and selection of papers |
| --- | --- |
| **Date:** | Friday, 19 July 2019 |
| **Databases searched:** | 1. EMBASE (n=1226)  2. MEDLINE (n=1169)  3. Web of Science (n=6192)  4. SCOPUS (n=1550)  5. COCHRANE LIBRARY (n=128)  6. Global Health – EBSCOHost (n=1588)  7. JSTOR (n=552) |

**1. EMBASE (*n*=1226)**

Final search string


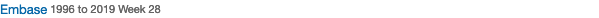


| [**# ▲**](https://ovidsp.dc2.ovid.com/sp-3.33.0b/ovidweb.cgi?&S=GOKOFPNGKMEBOMFFIPCKOHOGGDDHAA00&Sort+Sets=descending) | **Searches** | **Results** | **Type** | **Actions** |
| --- | --- | --- | --- | --- |
| 1 | Universal health coverage.ti,ab,kw. | 1879 | Advanced | [Display Results](https://ovidsp.dc2.ovid.com/sp-3.33.0b/ovidweb.cgi?&S=GOKOFPNGKMEBOMFFIPCKOHOGGDDHAA00&SELECT=S.sh%7c&R=1&Process+Action=display)  [More](https://ovidsp.dc2.ovid.com/sp-3.33.0b/ovidweb.cgi?S=GOKOFPNGKMEBOMFFIPCKOHOGGDDHAA00&Main+Search+Page=1) |
| 2 | Universal health insurance/ | 20 | Advanced | [Display Results](https://ovidsp.dc2.ovid.com/sp-3.33.0b/ovidweb.cgi?&S=GOKOFPNGKMEBOMFFIPCKOHOGGDDHAA00&SELECT=S.sh%7c&R=2&Process+Action=display)  [More](https://ovidsp.dc2.ovid.com/sp-3.33.0b/ovidweb.cgi?S=GOKOFPNGKMEBOMFFIPCKOHOGGDDHAA00&Main+Search+Page=1) |
| 3 | health coverage.mp. | 2888 | Advanced | [Display Results](https://ovidsp.dc2.ovid.com/sp-3.33.0b/ovidweb.cgi?&S=GOKOFPNGKMEBOMFFIPCKOHOGGDDHAA00&SELECT=S.sh%7c&R=3&Process+Action=display)  [More](https://ovidsp.dc2.ovid.com/sp-3.33.0b/ovidweb.cgi?S=GOKOFPNGKMEBOMFFIPCKOHOGGDDHAA00&Main+Search+Page=1) |
| 4 | uhc.mp. | 1162 | Advanced | [Display Results](https://ovidsp.dc2.ovid.com/sp-3.33.0b/ovidweb.cgi?&S=GOKOFPNGKMEBOMFFIPCKOHOGGDDHAA00&SELECT=S.sh%7c&R=4&Process+Action=display)  [More](https://ovidsp.dc2.ovid.com/sp-3.33.0b/ovidweb.cgi?S=GOKOFPNGKMEBOMFFIPCKOHOGGDDHAA00&Main+Search+Page=1) |
| 5 | couverture sante universelle.af. | 3 | Advanced | [Display Results](https://ovidsp.dc2.ovid.com/sp-3.33.0b/ovidweb.cgi?&S=GOKOFPNGKMEBOMFFIPCKOHOGGDDHAA00&SELECT=S.sh%7c&R=5&Process+Action=display)  [More](https://ovidsp.dc2.ovid.com/sp-3.33.0b/ovidweb.cgi?S=GOKOFPNGKMEBOMFFIPCKOHOGGDDHAA00&Main+Search+Page=1) |
| 6 | Universal health*.af. | 4332 | Advanced | [Display Results](https://ovidsp.dc2.ovid.com/sp-3.33.0b/ovidweb.cgi?&S=GOKOFPNGKMEBOMFFIPCKOHOGGDDHAA00&SELECT=S.sh%7c&R=6&Process+Action=display)  [More](https://ovidsp.dc2.ovid.com/sp-3.33.0b/ovidweb.cgi?S=GOKOFPNGKMEBOMFFIPCKOHOGGDDHAA00&Main+Search+Page=1) |
| 7 | universal coverage.af. | 1619 | Advanced | [Display Results](https://ovidsp.dc2.ovid.com/sp-3.33.0b/ovidweb.cgi?&S=GOKOFPNGKMEBOMFFIPCKOHOGGDDHAA00&SELECT=S.sh%7c&R=7&Process+Action=display)  [More](https://ovidsp.dc2.ovid.com/sp-3.33.0b/ovidweb.cgi?S=GOKOFPNGKMEBOMFFIPCKOHOGGDDHAA00&Main+Search+Page=1) |
| 8 | exp Africa/ | 255894 | Advanced | [Display Results](https://ovidsp.dc2.ovid.com/sp-3.33.0b/ovidweb.cgi?&S=GOKOFPNGKMEBOMFFIPCKOHOGGDDHAA00&SELECT=S.sh%7c&R=8&Process+Action=display)  [More](https://ovidsp.dc2.ovid.com/sp-3.33.0b/ovidweb.cgi?S=GOKOFPNGKMEBOMFFIPCKOHOGGDDHAA00&Main+Search+Page=1) |
| 9 | (Algeria or Algerie or Angola or Benin or Botswana or Burkina Faso or Burundi or Cameroon or Cameroun or Cape Verde or Cabo Verde or Cap-vert or Central African Republic or Republique centrafricaine or Chad or Tchad or Comoros or Comores or Democratic Republic of Congo or Republique democratique du congo or Republic of Congo or Republique du congo or Cote d'Ivoire or Djibouti or Egypt or Egypte or Equatorial Guinea or Guinee equatoriale or Eritrea or Erythree or Ethiopia or Ethiopie or Gabon or Gambia or Gambie or Ghana or Guinea or Guinee or Guinea Bissau or Guinee-bissau or Kenya or Lesotho or Liberia or Libya or Libye or Madagascar or Malawi or Mali or Mauritania or Mauritianie or Mauritius or Maurice or Morocco or Maroc or Mozambique or Namibia or Namibie or Niger or Nigeria or Reunion or Rwanda or (Sao Tome and Principe) or Sao Tome-et-Principe or Senegal or Seychelles or Sierra Leone or Somalia or Somalie or South Africa or Afrique du sud or South Sudan or Soudan du sud or Sudan or Soudan or Swaziland or Tanzania or Tanzanie or Togo or Tunisia or Tunisie or Uganda or Ouganda or Zambia or Zambie or Zimbabwe).af. | 645133 | Advanced | [Display Results](https://ovidsp.dc2.ovid.com/sp-3.33.0b/ovidweb.cgi?&S=GOKOFPNGKMEBOMFFIPCKOHOGGDDHAA00&SELECT=S.sh%7c&R=9&Process+Action=display)  [More](https://ovidsp.dc2.ovid.com/sp-3.33.0b/ovidweb.cgi?S=GOKOFPNGKMEBOMFFIPCKOHOGGDDHAA00&Main+Search+Page=1) |
| 10 | 1 or 2 or 3 or 4 or 5 or 6 or 7 | 7466 | Advanced | [Display Results](https://ovidsp.dc2.ovid.com/sp-3.33.0b/ovidweb.cgi?&S=GOKOFPNGKMEBOMFFIPCKOHOGGDDHAA00&SELECT=S.sh%7c&R=10&Process+Action=display)  [More](https://ovidsp.dc2.ovid.com/sp-3.33.0b/ovidweb.cgi?S=GOKOFPNGKMEBOMFFIPCKOHOGGDDHAA00&Main+Search+Page=1) |
| 11 | 8 or 9 | 672362 | Advanced | [Display Results](https://ovidsp.dc2.ovid.com/sp-3.33.0b/ovidweb.cgi?&S=GOKOFPNGKMEBOMFFIPCKOHOGGDDHAA00&SELECT=S.sh%7c&R=11&Process+Action=display)  [More](https://ovidsp.dc2.ovid.com/sp-3.33.0b/ovidweb.cgi?S=GOKOFPNGKMEBOMFFIPCKOHOGGDDHAA00&Main+Search+Page=1) |
| 12 | 10 and 11 | 1226 | Advanced | [Display Results](https://ovidsp.dc2.ovid.com/sp-3.33.0b/ovidweb.cgi?&S=GOKOFPNGKMEBOMFFIPCKOHOGGDDHAA00&SELECT=S.sh%7c&R=12&Process+Action=display)  [More](https://ovidsp.dc2.ovid.com/sp-3.33.0b/ovidweb.cgi?S=GOKOFPNGKMEBOMFFIPCKOHOGGDDHAA00&Main+Search+Page=1) |

**2. MEDLINE (*n*=1169)**

Final search string


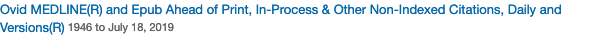


| **#**[**▲**](https://ovidsp.dc2.ovid.com/sp-3.33.0b/ovidweb.cgi?&S=PIHHFPCHEKEBOMOFJPCKEGBHHJCAAA00&Sort+Sets=descending) | **Searches** | **Results** | **Type** | **Actions** |
| --- | --- | --- | --- | --- |
| 1 | Universal health insurance/ | 3258 | Advanced | [Display Results](https://ovidsp.dc2.ovid.com/sp-3.33.0b/ovidweb.cgi?&S=PIHHFPCHEKEBOMOFJPCKEGBHHJCAAA00&SELECT=S.sh%7c&R=1&Process+Action=display)  [More](https://ovidsp.dc2.ovid.com/sp-3.33.0b/ovidweb.cgi?S=PIHHFPCHEKEBOMOFJPCKEGBHHJCAAA00&Main+Search+Page=1) |
| 2 | Universal health coverage.ti,ab,kw. | 1838 | Advanced | [Display Results](https://ovidsp.dc2.ovid.com/sp-3.33.0b/ovidweb.cgi?&S=PIHHFPCHEKEBOMOFJPCKEGBHHJCAAA00&SELECT=S.sh%7c&R=2&Process+Action=display)  [More](https://ovidsp.dc2.ovid.com/sp-3.33.0b/ovidweb.cgi?S=PIHHFPCHEKEBOMOFJPCKEGBHHJCAAA00&Main+Search+Page=1) |
| 3 | health coverage.af. | 2879 | Advanced | [Display Results](https://ovidsp.dc2.ovid.com/sp-3.33.0b/ovidweb.cgi?&S=PIHHFPCHEKEBOMOFJPCKEGBHHJCAAA00&SELECT=S.sh%7c&R=3&Process+Action=display)  [More](https://ovidsp.dc2.ovid.com/sp-3.33.0b/ovidweb.cgi?S=PIHHFPCHEKEBOMOFJPCKEGBHHJCAAA00&Main+Search+Page=1) |
| 4 | uhc.af. | 1344 | Advanced | [Display Results](https://ovidsp.dc2.ovid.com/sp-3.33.0b/ovidweb.cgi?&S=PIHHFPCHEKEBOMOFJPCKEGBHHJCAAA00&SELECT=S.sh%7c&R=4&Process+Action=display)  [More](https://ovidsp.dc2.ovid.com/sp-3.33.0b/ovidweb.cgi?S=PIHHFPCHEKEBOMOFJPCKEGBHHJCAAA00&Main+Search+Page=1) |
| 5 | universal coverage.af. | 1467 | Advanced | [Display Results](https://ovidsp.dc2.ovid.com/sp-3.33.0b/ovidweb.cgi?&S=PIHHFPCHEKEBOMOFJPCKEGBHHJCAAA00&SELECT=S.sh%7c&R=5&Process+Action=display)  [More](https://ovidsp.dc2.ovid.com/sp-3.33.0b/ovidweb.cgi?S=PIHHFPCHEKEBOMOFJPCKEGBHHJCAAA00&Main+Search+Page=1) |
| 6 | Universal health*.af. | 6056 | Advanced | [Display Results](https://ovidsp.dc2.ovid.com/sp-3.33.0b/ovidweb.cgi?&S=PIHHFPCHEKEBOMOFJPCKEGBHHJCAAA00&SELECT=S.sh%7c&R=6&Process+Action=display)  [More](https://ovidsp.dc2.ovid.com/sp-3.33.0b/ovidweb.cgi?S=PIHHFPCHEKEBOMOFJPCKEGBHHJCAAA00&Main+Search+Page=1) |
| 7 | couverture sante universelle.af. | 8 | Advanced | [Display Results](https://ovidsp.dc2.ovid.com/sp-3.33.0b/ovidweb.cgi?&S=PIHHFPCHEKEBOMOFJPCKEGBHHJCAAA00&SELECT=S.sh%7c&R=7&Process+Action=display)  [More](https://ovidsp.dc2.ovid.com/sp-3.33.0b/ovidweb.cgi?S=PIHHFPCHEKEBOMOFJPCKEGBHHJCAAA00&Main+Search+Page=1) |
| 8 | exp Africa/ | 250509 | Advanced | [Display Results](https://ovidsp.dc2.ovid.com/sp-3.33.0b/ovidweb.cgi?&S=PIHHFPCHEKEBOMOFJPCKEGBHHJCAAA00&SELECT=S.sh%7c&R=8&Process+Action=display)  [More](https://ovidsp.dc2.ovid.com/sp-3.33.0b/ovidweb.cgi?S=PIHHFPCHEKEBOMOFJPCKEGBHHJCAAA00&Main+Search+Page=1) |
| 9 | (Algeria or Algerie or Angola or Benin or Botswana or Burkina Faso or Burundi or Cameroon or Cameroun or Cape Verde or Cabo Verde or Cap-vert or Central African Republic or Republique centrafricaine or Chad or Tchad or Comoros or Comores or Democratic Republic of Congo or Republique democratique du congo or Republic of Congo or Republique du congo or Cote d'Ivoire or Djibouti or Egypt or Egypte or Equatorial Guinea or Guinee equatoriale or Eritrea or Erythree or Ethiopia or Ethiopie or Gabon or Gambia or Gambie or Ghana or Guinea or Guinee or Guinea Bissau or Guinee-bissau or Kenya or Lesotho or Liberia or Libya or Libye or Madagascar or Malawi or Mali or Mauritania or Mauritianie or Mauritius or Maurice or Morocco or Maroc or Mozambique or Namibia or Namibie or Niger or Nigeria or Reunion or Rwanda or (Sao Tome and Principe) or Sao Tome-et-Principe or Senegal or Seychelles or Sierra Leone or Somalia or Somalie or South Africa or Afrique du sud or South Sudan or Soudan du sud or Sudan or Soudan or Swaziland or Tanzania or Tanzanie or Togo or Tunisia or Tunisie or Uganda or Ouganda or Zambia or Zambie or Zimbabwe).af. | 743400 | Advanced | [Display Results](https://ovidsp.dc2.ovid.com/sp-3.33.0b/ovidweb.cgi?&S=PIHHFPCHEKEBOMOFJPCKEGBHHJCAAA00&SELECT=S.sh%7c&R=9&Process+Action=display)  [More](https://ovidsp.dc2.ovid.com/sp-3.33.0b/ovidweb.cgi?S=PIHHFPCHEKEBOMOFJPCKEGBHHJCAAA00&Main+Search+Page=1) |
| 10 | 1 or 2 or 3 or 4 or 5 or 6 or 7 | 8826 | Advanced | [Display Results](https://ovidsp.dc2.ovid.com/sp-3.33.0b/ovidweb.cgi?&S=PIHHFPCHEKEBOMOFJPCKEGBHHJCAAA00&SELECT=S.sh%7c&R=10&Process+Action=display)  [More](https://ovidsp.dc2.ovid.com/sp-3.33.0b/ovidweb.cgi?S=PIHHFPCHEKEBOMOFJPCKEGBHHJCAAA00&Main+Search+Page=1) |
| 11 | 8 or 9 | 773620 | Advanced | [Display Results](https://ovidsp.dc2.ovid.com/sp-3.33.0b/ovidweb.cgi?&S=PIHHFPCHEKEBOMOFJPCKEGBHHJCAAA00&SELECT=S.sh%7c&R=11&Process+Action=display)  [More](https://ovidsp.dc2.ovid.com/sp-3.33.0b/ovidweb.cgi?S=PIHHFPCHEKEBOMOFJPCKEGBHHJCAAA00&Main+Search+Page=1) |
| 12 | 10 and 11 | 1169 | Advanced | [Display Results](https://ovidsp.dc2.ovid.com/sp-3.33.0b/ovidweb.cgi?&S=PIHHFPCHEKEBOMOFJPCKEGBHHJCAAA00&SELECT=S.sh%7c&R=12&Process+Action=display)  [More](https://ovidsp.dc2.ovid.com/sp-3.33.0b/ovidweb.cgi?S=PIHHFPCHEKEBOMOFJPCKEGBHHJCAAA00&Main+Search+Page=1) |

**3. Web of Science (*n*=6192)**

Final search string

Web of Science Core Collection

All years (1900 – 2019)

| Set | Results | Topic/Combination |
| --- | --- | --- |
| # 12 | [6,192](http://apps.webofknowledge.com/summary.do;jsessionid=3F4EC15A2312C47E075CA0E37161087D?product=WOS&doc=1&qid=13&SID=F6xD6okMdSLBocHApr4&search_mode=CombineSearches&update_back2search_link_param=yes) | #11 AND #10  Indexes=SCI-EXPANDED, SSCI, A&HCI, CPCI-S, CPCI-SSH, BKCI-S, BKCI-SSH, ESCI, CCR-EXPANDED, IC Timespan=All years |
| # 11 | [831,976](http://apps.webofknowledge.com/summary.do;jsessionid=3F4EC15A2312C47E075CA0E37161087D?product=WOS&doc=1&qid=12&SID=F6xD6okMdSLBocHApr4&search_mode=CombineSearches&update_back2search_link_param=yes) | #9 OR #8  Indexes=SCI-EXPANDED, SSCI, A&HCI, CPCI-S, CPCI-SSH, BKCI-S, BKCI-SSH, ESCI, CCR-EXPANDED, IC Timespan=All years |
| # 10 | [54,692](http://apps.webofknowledge.com/summary.do;jsessionid=3F4EC15A2312C47E075CA0E37161087D?product=WOS&doc=1&qid=11&SID=F6xD6okMdSLBocHApr4&search_mode=CombineSearches&update_back2search_link_param=yes) | #7 OR #6 OR #5 OR #4 OR #3 OR #2 OR #1  Indexes=SCI-EXPANDED, SSCI, A&HCI, CPCI-S, CPCI-SSH, BKCI-S, BKCI-SSH, ESCI, CCR-EXPANDED, IC Timespan=All years |
| # 9 | [714,390](http://apps.webofknowledge.com/summary.do;jsessionid=3F4EC15A2312C47E075CA0E37161087D?product=WOS&doc=1&qid=9&SID=F6xD6okMdSLBocHApr4&search_mode=GeneralSearch&update_back2search_link_param=yes) | **TOPIC:** (Algeria or Algerie or Angola or Benin or Botswana or Burkina Faso or Burundi or Cameroon or Cameroun or Cape Verde or Cabo Verde or Cap-vert or Central African Republic or Republique centrafricaine or Chad or Tchad or Comoros or Comores or Democratic Republic of Congo or Republique democratique du congo or Republic of Congo or Republique du congo or Cote d'Ivoire or Djibouti or Egypt or Egypte or Equatorial Guinea or Guinee equatoriale or Eritrea or Erythree or Ethiopia or Ethiopie or Gabon or Gambia or Gambie or Ghana or Guinea or Guinee or Guinea Bissau or Guinee-bissau or Kenya or Lesotho or Liberia or Libya or Libye or Madagascar or Malawi or Mali or Mauritania or Mauritianie or Mauritius or Maurice or Morocco or Maroc or Mozambique or Namibia or Namibie or Niger or Nigeria or Reunion or Rwanda or (Sao Tome and Principe) or Sao Tome-et-Principe or Senegal or Seychelles or Sierra Leone or Somalia or Somalie or South Africa or Afrique du sud or South Sudan or Soudan du sud or Sudan or Soudan or Swaziland or Tanzania or Tanzanie or Togo or Tunisia or Tunisie or Uganda or Ouganda or Zambia or Zambie or Zimbabwe)  Indexes=SCI-EXPANDED, SSCI, A&HCI, CPCI-S, CPCI-SSH, BKCI-S, BKCI-SSH, ESCI, CCR-EXPANDED, IC Timespan=All years |
| # 8 | [321,645](http://apps.webofknowledge.com/summary.do;jsessionid=3F4EC15A2312C47E075CA0E37161087D?product=WOS&doc=1&qid=8&SID=F6xD6okMdSLBocHApr4&search_mode=GeneralSearch&update_back2search_link_param=yes) | **TOPIC:** (africa)  Indexes=SCI-EXPANDED, SSCI, A&HCI, CPCI-S, CPCI-SSH, BKCI-S, BKCI-SSH, ESCI, CCR-EXPANDED, IC Timespan=All years |
| # 7 | [8](http://apps.webofknowledge.com/summary.do;jsessionid=3F4EC15A2312C47E075CA0E37161087D?product=WOS&doc=1&qid=7&SID=F6xD6okMdSLBocHApr4&search_mode=GeneralSearch&update_back2search_link_param=yes) | **TOPIC:** (couverture sante universelle)  Indexes=SCI-EXPANDED, SSCI, A&HCI, CPCI-S, CPCI-SSH, BKCI-S, BKCI-SSH, ESCI, CCR-EXPANDED, IC Timespan=All years |
| # 6 | [1,123](http://apps.webofknowledge.com/summary.do;jsessionid=3F4EC15A2312C47E075CA0E37161087D?product=WOS&doc=1&qid=6&SID=F6xD6okMdSLBocHApr4&search_mode=GeneralSearch&update_back2search_link_param=yes) | **TOPIC:** (uhc)  Indexes=SCI-EXPANDED, SSCI, A&HCI, CPCI-S, CPCI-SSH, BKCI-S, BKCI-SSH, ESCI, CCR-EXPANDED, IC Timespan=All years |
| # 5 | [6,457](http://apps.webofknowledge.com/summary.do;jsessionid=3F4EC15A2312C47E075CA0E37161087D?product=WOS&doc=1&qid=5&SID=F6xD6okMdSLBocHApr4&search_mode=GeneralSearch&update_back2search_link_param=yes) | **TOPIC:** (universal coverage)  Indexes=SCI-EXPANDED, SSCI, A&HCI, CPCI-S, CPCI-SSH, BKCI-S, BKCI-SSH, ESCI, CCR-EXPANDED, IC Timespan=All years |
| # 4 | [35,633](http://apps.webofknowledge.com/summary.do;jsessionid=3F4EC15A2312C47E075CA0E37161087D?product=WOS&doc=1&qid=4&SID=F6xD6okMdSLBocHApr4&search_mode=GeneralSearch&update_back2search_link_param=yes) | **TOPIC:** (health coverage)  Indexes=SCI-EXPANDED, SSCI, A&HCI, CPCI-S, CPCI-SSH, BKCI-S, BKCI-SSH, ESCI, CCR-EXPANDED, IC Timespan=All years |
| # 3 | [2,611](http://apps.webofknowledge.com/summary.do;jsessionid=3F4EC15A2312C47E075CA0E37161087D?product=WOS&doc=1&qid=3&SID=F6xD6okMdSLBocHApr4&search_mode=GeneralSearch&update_back2search_link_param=yes) | **TOPIC:** (Universal health insurance/)  Indexes=SCI-EXPANDED, SSCI, A&HCI, CPCI-S, CPCI-SSH, BKCI-S, BKCI-SSH, ESCI, CCR-EXPANDED, IC Timespan=All years |
| # 2 | [20,665](http://apps.webofknowledge.com/summary.do;jsessionid=9EC617E6568E14C0AA235707DA08F1B0?product=WOS&doc=1&qid=2&SID=F6xD6okMdSLBocHApr4&search_mode=GeneralSearch&update_back2search_link_param=yes) | **TOPIC:** (Universal health*)  Indexes=SCI-EXPANDED, SSCI, A&HCI, CPCI-S, CPCI-SSH, BKCI-S, BKCI-SSH, ESCI, CCR-EXPANDED, IC Timespan=All years |
| # 1 | [4,351](http://apps.webofknowledge.com/summary.do;jsessionid=9EC617E6568E14C0AA235707DA08F1B0?product=WOS&doc=1&qid=1&SID=F6xD6okMdSLBocHApr4&search_mode=GeneralSearch&update_back2search_link_param=yes) | **TOPIC:** (Universal health coverage)  Indexes=SCI-EXPANDED, SSCI, A&HCI, CPCI-S, CPCI-SSH, BKCI-S, BKCI-SSH, ESCI, CCR-EXPANDED, IC Timespan=All years |

**4. SCOPUS (*n*=1550)**

Final search string

( ( TITLE-ABS-KEY ( universal  AND health  AND coverage ) )  OR  ( TITLE-ABS-KEY ( universal  AND health* ) )  OR  ( TITLE-ABS-KEY ( "Universal health*" ) )  OR  ( TITLE-ABS-KEY ( "universal health coverage" ) )  OR  ( TITLE-ABS-KEY ( "couverture sante universelle" ) )  OR  ( TITLE-ABS-KEY ( "health coverage" ) )  OR  ( TITLE-ABS-KEY ( uhc ) )  OR  ( TITLE-ABS-KEY ( "universal coverage" ) )  OR  ( TITLE-ABS-KEY ( "universal health" ) ) )  AND  ( ( TITLE-ABS-KEY ( africa ) )  OR  ( TITLE-ABS-KEY ( algeria  OR  algerie  OR  angola  OR  benin  OR  botswana  OR  burkina  AND faso  OR  burundi  OR  cameroon  OR  cameroun  OR  cape  AND verde  OR  cabo  AND verde  OR  cap-vert  OR  central  AND african  AND republic  OR  republique  AND centrafricaine  OR  chad  OR  tchad  OR  comoros  OR  comores ) )  OR  ( TITLE-ABS-KEY ( algeria  OR  algerie  OR  angola  OR  benin  OR  botswana  OR  burkina  AND faso  OR  burundi  OR  cameroon  OR  cameroun  OR  cape  AND verde  OR  cabo  AND verde  OR  cap-vert  OR  "Central African Republic"  OR  republique  AND centrafricaine  OR  chad  OR  tchad  OR  comoros  OR  comores ) )  OR  ( TITLE-ABS-KEY ( ethiopia  OR  ethiopie  OR  gabon  OR  gambia  OR  gambie  OR  ghana  OR  guinea  OR  guinee  OR  guinea  AND bissau  OR  guinee-bissau  OR  kenya  OR  lesotho  OR  liberia  OR  libya  OR  libye  OR  madagascar  OR  malawi  OR  mali  OR  mauritania ) )  OR  ( TITLE-ABS-KEY ( mauritianie  OR  mauritius  OR  maurice  OR  morocco  OR  maroc  OR  mozambique  OR  namibia  OR  namibie  OR  niger  OR  nigeria  OR  reunion  OR  rwanda  OR  ( sao  AND tome  AND  principe )  OR  sao  AND tome-et-principe  OR  senegal  OR  seychelles  OR  sierra  AND leone ) )  OR  ( TITLE-ABS-KEY ( somalia  OR  somalie  OR  south  AND africa  OR  afrique  AND du  AND sud  OR  south  AND sudan  OR  soudan  AND du  AND sud  OR  sudan  OR  soudan  OR  swaziland  OR  tanzania  OR  tanzanie  OR  togo  OR  tunisia  OR  tunisie  OR  uganda  OR  ouganda  OR  zambia  OR  zambie  OR  zimbabwe ) ) )

**5. COCHRANE LIBRARY (*n*=128)**

Final search string, n=123 + 5 = 128

1. 123 Cochrane Reviews matching:

Universal Health Coverage in Title Abstract Keyword OR universal health insurance in Title Abstract Keyword OR universal coverage in Title Abstract Keyword OR universal health in Title Abstract Keyword OR couverture sante universelle in Title Abstract Keyword - (Word variations have been searched)

[Cochrane Database of Systematic Reviews](https://www.cochranelibrary.com/)

Issue 7 of 12, July 2019

AND

1. 5 Trials matching:

MeSH descriptor: [Universal Health Insurance] explode all trees

[*Cochrane* Central Register of Controlled Trials](https://www.cochranelibrary.com/)

Issue 7 of 12, July 2019

**6. Global Health – EBSCOHost (*n*=1588)**

Final search string, n=1588

| [**Search ID#**](javascript:__doPostBack('ctl00$ctl00$MainContentArea$MainContentArea$historyControl$ReorderHistoryLink','')) | **Search Terms** | **Search Options** | **Actions** |
| --- | --- | --- | --- |
| S12 | S8 AND S11 | Search modes - Boolean/Phrase | [View Results](javascript:__doPostBack('ctl00$ctl00$MainContentArea$MainContentArea$historyControl$HistoryRepeater$ctl00$linkResults','')) (1,588)  [View Details](javascript:showShDetails(%22ctl00_ctl00_MainContentArea_MainContentArea_historyControl_ctrlPopup%22,%20%22S12%22);)  [Edit](https://web.a.ebscohost.com/Legacy/Views/UserControls/Ehost/) |
| S11 | S9 OR S10 | Search modes - Boolean/Phrase | [View Results](javascript:__doPostBack('ctl00$ctl00$MainContentArea$MainContentArea$historyControl$HistoryRepeater$ctl01$linkResults','')) (294,751)  [View Details](javascript:showShDetails(%22ctl00_ctl00_MainContentArea_MainContentArea_historyControl_ctrlPopup%22,%20%22S11%22);)  [Edit](https://web.a.ebscohost.com/Legacy/Views/UserControls/Ehost/) |
| S10 | africa | Search modes - Boolean/Phrase | [View Results](javascript:__doPostBack('ctl00$ctl00$MainContentArea$MainContentArea$historyControl$HistoryRepeater$ctl02$linkResults','')) (215,801)  [View Details](javascript:showShDetails(%22ctl00_ctl00_MainContentArea_MainContentArea_historyControl_ctrlPopup%22,%20%22S10%22);)  [Edit](https://web.a.ebscohost.com/Legacy/Views/UserControls/Ehost/) |
| S9 | Algeria or Algerie or Angola or Benin or Botswana or Burkina Faso or Burundi or Cameroon or Cameroun or Cape Verde or Cabo Verde or Cap-vert or Central African Republic or Republique centrafricaine or Chad or Tchad or Comoros or Comores or Democratic Republic of Congo or Republique democratique du congo or Republic of Congo or Republique du congo or Cote d'Ivoire or Djibouti or Egypt or Egypte or Equatorial Guinea or Guinee equatoriale or Eritrea or Erythree or Ethiopia or Ethiopie or Gabon or G [...](javascript:showHistoryTerm('ctl00_ctl00_MainContentArea_MainContentArea_historyControl_HistoryRepeater_ctl03_ellipsis',true)) | Search modes - Boolean/Phrase | [View Results](javascript:__doPostBack('ctl00$ctl00$MainContentArea$MainContentArea$historyControl$HistoryRepeater$ctl03$linkResults','')) (280,029)  [View Details](javascript:showShDetails(%22ctl00_ctl00_MainContentArea_MainContentArea_historyControl_ctrlPopup%22,%20%22S9%22);)  [Edit](https://web.a.ebscohost.com/Legacy/Views/UserControls/Ehost/) |
| S8 | S1 OR S2 OR S3 OR S4 OR S5 OR S6 OR S7 | Search modes - Boolean/Phrase | [View Results](javascript:__doPostBack('ctl00$ctl00$MainContentArea$MainContentArea$historyControl$HistoryRepeater$ctl04$linkResults','')) (6,953)  [View Details](javascript:showShDetails(%22ctl00_ctl00_MainContentArea_MainContentArea_historyControl_ctrlPopup%22,%20%22S8%22);)  [Edit](https://web.a.ebscohost.com/Legacy/Views/UserControls/Ehost/) |
| S7 | couverture sante universelle | Search modes - Boolean/Phrase | [View Results](javascript:__doPostBack('ctl00$ctl00$MainContentArea$MainContentArea$historyControl$HistoryRepeater$ctl05$linkResults','')) (1)  [View Details](javascript:showShDetails(%22ctl00_ctl00_MainContentArea_MainContentArea_historyControl_ctrlPopup%22,%20%22S7%22);)  [Edit](https://web.a.ebscohost.com/Legacy/Views/UserControls/Ehost/) |
| S6 | universal coverage | Search modes - Boolean/Phrase | [View Results](javascript:__doPostBack('ctl00$ctl00$MainContentArea$MainContentArea$historyControl$HistoryRepeater$ctl06$linkResults','')) (2,093)  [View Details](javascript:showShDetails(%22ctl00_ctl00_MainContentArea_MainContentArea_historyControl_ctrlPopup%22,%20%22S6%22);)  [Edit](https://web.a.ebscohost.com/Legacy/Views/UserControls/Ehost/) |
| S5 | uhc | Search modes - Boolean/Phrase | [View Results](javascript:__doPostBack('ctl00$ctl00$MainContentArea$MainContentArea$historyControl$HistoryRepeater$ctl07$linkResults','')) (486)  [View Details](javascript:showShDetails(%22ctl00_ctl00_MainContentArea_MainContentArea_historyControl_ctrlPopup%22,%20%22S5%22);)  [Edit](https://web.a.ebscohost.com/Legacy/Views/UserControls/Ehost/) |
| S4 | health coverage | Search modes - Boolean/Phrase | [View Results](javascript:__doPostBack('ctl00$ctl00$MainContentArea$MainContentArea$historyControl$HistoryRepeater$ctl08$linkResults','')) (4,733)  [View Details](javascript:showShDetails(%22ctl00_ctl00_MainContentArea_MainContentArea_historyControl_ctrlPopup%22,%20%22S4%22);)  [Edit](https://web.a.ebscohost.com/Legacy/Views/UserControls/Ehost/) |
| S3 | Universal health insurance/ | Search modes - Boolean/Phrase | [View Results](javascript:__doPostBack('ctl00$ctl00$MainContentArea$MainContentArea$historyControl$HistoryRepeater$ctl09$linkResults','')) (344)  [View Details](javascript:showShDetails(%22ctl00_ctl00_MainContentArea_MainContentArea_historyControl_ctrlPopup%22,%20%22S3%22);)  [Edit](https://web.a.ebscohost.com/Legacy/Views/UserControls/Ehost/) |
| S2 | Universal health* | Search modes - Boolean/Phrase | [View Results](javascript:__doPostBack('ctl00$ctl00$MainContentArea$MainContentArea$historyControl$HistoryRepeater$ctl10$linkResults','')) (2,995)  [View Details](javascript:showShDetails(%22ctl00_ctl00_MainContentArea_MainContentArea_historyControl_ctrlPopup%22,%20%22S2%22);)  [Edit](https://web.a.ebscohost.com/Legacy/Views/UserControls/Ehost/) |
| S1 | Universal health coverage | Search modes - Boolean/Phrase | [View Results](javascript:__doPostBack('ctl00$ctl00$MainContentArea$MainContentArea$historyControl$HistoryRepeater$ctl11$linkResults','')) (1,455)  [View Details](javascript:showShDetails(%22ctl00_ctl00_MainContentArea_MainContentArea_historyControl_ctrlPopup%22,%20%22S1%22);) |

**7. JSTOR (*n*=552)**

("universal health coverage" OR "universal coverage") AND Africa

n=552
