# Supplementary material for: Health financing for universal health coverage in Sub-Saharan Africa: a systematic review
Source: Glob Health Res Policy. 2021 Mar 1;6:8. doi: 10.1186/s41256-021-00190-7 (PMC7916997; doi:10.1186/s41256-021-00190-7)
Supplement: Supplementary file 4 — Additional file 4. [file 41256_2021_190_MOESM4_ESM.docx]

## Additional file 4. Table of health financing mechanisms reported within the systematic review results (n=39)

| Count | Author (s) | Setting | National Health Insurance (NHI) | Tax-based Financing | Community-based Health Insurance (CBHI) | Out-of-pocket payments (OOPs) | External (donor) Financing | Other Financing Mechanisms |
| --- | --- | --- | --- | --- | --- | --- | --- | --- |
| 1 | Abiiro et al. | Malawi |  | x |  | x | x |  |
| 2 | Aregbeshola | Nigeria |  | x |  | x | x |  |
| 3 | Ataguba and McIntyre | South Africa | x |  |  | x |  |  |
| 4 | Awosusi et al. | Nigeria | x |  |  | x | x |  |
| 5 | Baine et al. | Uganda |  |  | x | x |  |  |
| 6 | Barasa et al. | Kenya | x |  |  | x | x |  |
| 7 | Barasa et al. | Kenya | x |  |  | x | x |  |
| 8 | Bertone et al. | Multiple in SSA* | x |  |  | x | x |  |
| 9 | Chemouni | Rwanda |  |  | x | x | x |  |
| 10 | Chilufya and Kamanga | Zambia | x | x |  |  | x |  |
| 11 | Chuma and Okungu | Kenya | x |  |  | x | x |  |
| 12 | Dieleman et al. | Multiple in SSA | x |  |  | x | x |  |
| 13 | Fusheini and Eyles | South Africa | x |  |  | x |  |  |
| 14 | Goeppel et al. | Multiple in SSA | x |  |  | x |  |  |
| 15 | Hafez | Nigeria |  | x |  | x | x |  |
| 16 | Hanlon et al. | Ethiopia | x |  | x | x |  |  |
| 17 | Lavers | Ethiopia | x |  | x | x | x |  |
| 18 | Lu et al. | Rwanda |  |  | x | x | x |  |
| 19 | Ly et al. | Multiple in SSA |  | x |  | x | x |  |
| 20 | Makinde et al. | Nigeria | x |  |  | x |  |  |
| 21 | Marten et al. | South Africa | x | x |  | x |  |  |
| 22 | McIntyre et al. | Multiple in SSA |  | x |  | x | x |  |
| 23 | McIntyre et al. | Multiple in SSA | x |  |  | x | x |  |
| 24 | McIntyre et al. | Multiple in SSA | x | x | x | x | x | x |
| 25 | Mulenga and Ataguba | Zambia | x | x |  | x | x |  |
| 26 | Munge et al. | Kenya |  |  |  | x | x | x |
| 27 | Okech and Lelegwe | Kenya | x |  |  |  | x |  |
| 28 | Okungu and McIntyre | Kenya | x |  |  |  | x | x |
| 29 | Okungu et al. | Kenya | x |  |  |  |  |  |
| 30 | Ota et al. | Multiple in SSA | x |  |  | x | x |  |
| 31 | Pettigrew and Mathauer | Multiple in SSA |  |  |  | x | x | x |
| 32 | Reeves et al. | Multiple in SSA |  | x |  | x | x |  |
| 33 | Sambo and Kirigia | Multiple in SSA | x | x |  |  | x |  |
| 34 | Sanogo et al. | Multiple in SSA | x |  |  | x |  |  |
| 35 | Ssennyonjo et al. | Uganda |  | x |  | x | x |  |
| 36 | Uzochukwu et al. | Nigeria | x | x | x | x | x |  |
| 37 | van den Heever | South Africa | x |  |  | x |  |  |
| 38 | Wang and Rosemberg | Tanzania | x |  |  | x | x |  |
| 39 | Wang et al. | Tanzania | x |  |  | x | x |  |
|  |  | Totals | 27 | 13 | 7 | 34 | 29 | 4 |

***Notes and some considerations:***

**SSA = Sub-Saharan Africa.*

***Out of 39 studies, 12 are cross-cutting - involve multiple countries in SSA; 27 are related to nine individual countries. OOPs are regressive forms of financing, least efficient and equitable. However, a majority still rely on OOPs. Finally, solely because a mechanism is not mentioned, does not mean it does not exist in the given country.*
