# Supplementary material for: Health financing for universal health coverage in Sub-Saharan Africa: a systematic review
Source: Glob Health Res Policy. 2021 Mar 1;6:8. doi: 10.1186/s41256-021-00190-7 (PMC7916997; doi:10.1186/s41256-021-00190-7)
Supplement: Supplementary file 5 — Additional file 5. [file 41256_2021_190_MOESM5_ESM.docx]

## Additional file 4. Comprehensive table of results showing the main characteristics

| Author (s) | Year | Study Objective | Population | Setting | Study Design | Some of the central recommendations | Quality Score |
| --- | --- | --- | --- | --- | --- | --- | --- |
| Abiiro et al. | 2014 | To explore how rural communities experience and define gaps in universal health coverage in Malawi, a country which endorses free access to an Essential Health Package as a means towards UHC. | Six rural communities in Malawi. A total of 127 community residents participated. | Thyolo and Chiradzulu, two rural districts in Southern Malawi | Qualitative: Cross-sectional Study; Descriptive/Exploratory | To move towards UHC in Malawi, the possibility of an effective public-private partnership needs to be explored, in order to harness the potentials of the private sector to complement the UHC efforts in the public sector. People-centred and health system responsive UHC reforms and a bottom-up approach is needed driven by local evidence reflecting context-specific needs. | High |
| Aregbeshola | 2018 | To propose the design and effective implementation of a non-contributory mechanism for health financing toward achieving UHC in Nigeria. | Nigerian population; estimated 186 million in 2016. | Nigeria | Descriptive/Exploratory; Perspective | Policy makers should consider adopting a tax-based, non-contributory, universal health financing system as the primary funding mechanism to accelerate progress toward UHC to extend coverage and access, given the country’s large poor, vulnerable and informal sector population. | High |
| Ataguba and McIntyre | 2017 | To assess the progressivity of each health financing mechanisms (direct and indirect taxes, OOPs and private health insurance) and overall progressivity of health financing in South Africa. | 27,665 households | South Africa | Quantitative; Cross-sectional study: household survey; Descriptive | This study contributes to policy-making, particularly in relation to the proposed NHI in South Africa, in the sense that it provides updated empirical evidence on the relative progressivity of alternative health financing mechanisms, and so health financing reforms can be tailored to policy-makers’ preferences drawing on this evidence base. | Medium |
| Awosusi et al. | 2015 | N/A | Nigerian population | Nigeria | Descriptive/Exploratory | The new government should creatively and aggressively explore innovative domestic financing despite attendant fiscal constraints. Tax avoidance and inefficient tax collection are major roadblocks that the new government should tackle to improve domestic revenue generation. | High |
| Baine et al. | 2018 | To describe Kisiizi Hospital Health Insurance scheme (KHHIS) and to document lessons learned and implications for UHC. | Enrolees and stakeholders of KHHIS | Kisiizi hospital, a faith-based hospital in South Western Uganda | Mixed methods (qualitative and quantitative): Descriptive cross-sectional study | Development of a health insurance scheme requires ample time and was not a one time-off process. It took KHHIS 21 years and positive progress has been registered. Resilience is success factor in the development of health insurance schemes. CBHI schemes similar to KHHIS may not be replicated directly in other areas with different cultural complexities. | High |
| Barasa et al. | 2018 | To develop a summary measure of UHC for Kenya and track the country’s progress between 2003 and 2013. | Three rounds of the Kenya Demographic and Health Survey (KDHS) data in 2003, 2007, 2013: in 2013, 31 079 (women) and 12 819 (men). Three rounds of the Kenya Household Expenditure and Utilisation Survey (KHHEUS) in 2003, 2007, 2013: in 2013, 152 566 individuals. | Kenya | Quantitative; Cross-sectional study: household survey | A formal analysis of the fiscal space for health in Kenya is required to identify the most feasible strategies to improve public financing for healthcare in Kenya. The country needs to reorient its health financing strategy away from a focus on contributory, voluntary health insurance, and instead recognise that increased tax funding is critical. | High |
| Barasa et al. | 2018 | To identify and describe the reforms undertaken by the National Hospital Insurance Fund and examine the implications for Kenya’s quest to achieve UHC. | National Hospital Insurance Fund members | Kenya | Qualitative; Descriptive assessment of reforms | As Kenya makes the difficult choices related to what services to provide and to whom and the extent of financial risk protection, it is important that such decisions ensure fairness and equity. | Medium |
| Bertone et al. | 2019 | To update the exploratory literature review conducted in 2012 to analyse the main themes and findings of recent literature focusing on health financing in fragile and conflict-affected settings (FCAS)*. | Populations in FCAS | FCAS | Qualitative; Descriptive/Exploratory literature review | Health financing reforms have the potential not only to allow progress towards UHC, but also to communicate political and social values. This is particularly important in fragile, post-conflict recovery settings where they potentially link to broader confidence-building processes. | Medium |
| Chemouni | 2018 | To explain how Rwanda, one of the poorest countries in the world, managed to achieve such performance by understanding the political drivers behind the CBHI design and implementation. | Enrolees of CBHI named "Mutuelles de santé" | Rwanda | Qualitative; Descriptive/Exploratory | The Rwandan experience is a powerful call for thinking harder on ways to improve enrolment, for example through innovative financing mechanisms such as saving associations to pay premiums or large donor funding, or through improving the quality of care. It also further highlights that myriad of independent, low enrolment, CBHI schemes, will probably not be enough to pursue UHC. | Medium |
| Chilufya and Kamanga | 2018 | To describe Zambia's trajectory to achieving UHC from the 1990s to date. To highlight some of the past institutional and financing reforms, achievements, gaps and challenges that the government is determined to address through an explicit transformational agenda launched in 2011. | Zambian population | Zambia | Qualitative; Descriptive/Exploratory | All aspects of the Zambian health system are being strengthened by using the primary health care approach with a view of shifting focus from curative to preventive community-based care. In addition to increased government funding, technical and allocative inefficiencies should be addressed to eliminate unnecessary waste and provide better services from available resources. This requires radical changes to strengthen health systems and increase coverage of equity-focused priority interventions as these are key for UHC. | Medium |
| Chuma and Okungu | 2011 | To assess the extent to which the Kenyan health financing system meets the key requirements for universal coverage, including income and risk cross-subsidisation. | Kenyan population | Kenya | Quantitative; analysis of health expenditure data | Some progress has been made towards addressing equity challenges, including reducing user fees at primary health care facilities and developing a health financing strategy, but universal coverage is unlikely to be achieved unless the country adopts a systemic approach to health financing reforms. Such an approach should be informed by the wider health system goals of equity and efficiency. | Medium |
| Dieleman et al.*  *Global Burden of Disease Health Financing Collaborator Network* | 2018 | To estimate future scenarios of health spending and pooled health spending through to 2400 using historical health financing data for 188 countries from 1995 to 2015. | 188 countries | Health spending data for 188 countries | Quantitative; modelling and health spending data analysis | It is important to recognise that increasing health spending is neither a necessary nor sufficient condition for improving UHC. Rather, ensuring a supply of additional pooled resources for UHC, alongside other important socio-political factors and policy levers, is likely to provide a strong foundation for equity focused, sustainable UHC programmes. | High |
| Fusheini and Eyles | 2016 | To explore the opportunities and challenges, of the district health system in achieving UHC in South Africa. | South African population of district health systems | South Africa | Qualitative; Descriptive/Exploratory | UHC through NHI implies a move towards equity of access and financial risk protection, especially, by which the most excluded and vulnerable populations rise to the same standards of health enjoyed by the more privileged in society. | Medium |
| Goeppel et al. | 2016 | To assess UHC for adults aged 50 years or older with chronic illness in China, Ghana, India, Mexico, the Russian Federation and South Africa. | 16 631 participants aged 50 years or older who had at least one diagnosed chronic condition from the WHO Study on Global Ageing and Adult Health (SAGE)  Ghana: 1327  South Africa: 1866 | China, Ghana, India, Mexico, the Russian Federation and South Africa | Quantitative; Cross-sectional study: household survey | The provision of UHC for older people with chronic conditions is particularly challenging for LMICs, especially given the ongoing epidemiological transition. It is crucial, therefore, that future health policies are tailored to the specific needs of older people. | High |
| Hafez | 2018 | To highlight constraints and opportunities for building a sustainable health financing system that accelerates progress towards UHC. | Nigerian population (approx. 182 million people) | Nigeria | Quantitative; Cross-sectional study: household survey | Moving forward, it would be important to understand why NHIS coverage has failed to move beyond the formal sector. In conjunction with the basic health care provision fund, a federal program, the health sector will need to continue strengthening core health financing functions including overall planning and budgeting, and system performance monitoring and evaluation. | Low |
| Hanlon et al. | 2019 | To propose context-relevant strategies for moving towards UHC for people with mental disorders in Ethiopia. | People with mental health disorders | Ethiopia | Qualitative; SWOT Analysis | Three main strategies are proposed for improving public sector performance as well as more equitable financing with respect to mental health care in Ethiopia: (1) increasing efficiency of use of existing resources, (2) increasing revenue for domestic sources and (3) increasing external financing. | High |
| Lavers | 2019 | To examine the political drivers of the adoption and evolution of state health insurance. | 28 actors in policymaking process | Ethiopia | Qualitative; Descriptive/Exploratory | CBHI and SHI must be merged since developing separate schemes would institutionalise a two-tier healthcare system, while CBHI cannot be financially sustainable on its own. For a government seeking to expand health access, there are two main options: remove user fees and finance healthcare through general revenues; or introduce insurance to reduce OOP spending. The choice of health insurance was the result not only of bureaucrats, but the paradigmatic ideas underpinning the political settlement. | High |
| Lu et al. | 2012 | To evaluate the impact of Mutuelles on achieving universal coverage of medical services and financial risk protection in its first eight years of implementation. | Rwandan general population, under-five children, and women with delivery | Rwanda | Quantitative; Cross-sectional study: household survey | The Rwanda experience offers valuable lessons to other LICs that are in a similarly challenging situation. The government played a crucial role through increased financial investment in the health sector, successful legislation of the entitlement of basic care for uninsured population, and an intensive nationwide campaign. | High |
| Ly et al. | 2017 | To review the trends from 1995 to the present and assess the feasibility of financing UHC in SSA now and by 2030. | Sub-Saharan African population | Sub-Saharan Africa | Quantitative; cross-sectional and longitudinal economic and health spending data | SSA countries and donors need to consider five options depending on the trajectory of their health financing prospects from domestic and donor sources. There is an opportunity now to support a much more efficient and equitable transition from donor assistance for health dependence to prepaid, pooled domestic financing. These options are not mutually exclusive, and solutions should take into consideration a mix of options. | Low |
| Makinde et al. | 2018 | To review the geographic and sectoral distribution of health facilities in Nigeria and discuss implications on the UHC strategy selected. | Nigerian population (approx. 184 million residents) | Nigeria | Quantitative; Cross-sectional study data | There has been a call to make health insurance compulsory with concrete steps taken on the amendment of the National Health Insurance Scheme Act passed by the Seventh National Assembly (awaiting presidential assent), as no country has achieved UHC based on voluntary participation in health insurance schemes. | Medium |
| Marten et al. | 2014 | To assess health systems and reforms towards UHC and consider these efforts and remaining challenges using a simple framework. | South African population (in focus for data extraction)  Brazil, the Russian Federation, India, China, and South Africa (BRICS) | South Africa | Qualitative; Descriptive/Exploratory | The most pressing problems are raising insufficient public spending; stewarding mixed private and public health systems; ensuring equity; meeting the demands for more human resources; managing changing demographics and disease burdens; and addressing the social determinants of health. Increases in public funding can be used to show how BRICS health ministries could accelerate progress to achieve UHC. | Medium |
| McIntyre et al. | 2017 | To explore potential targets for government spending on health to progress towards UHC. | Global and Sub-Saharan African population | Global and Sub-Saharan Africa | Quantitative; analysis of health expenditure data | LMICs will not make substantial progress towards universal health systems unless governments make concerted efforts to maximise their ‘available resources’. An explicit target for government expenditure on health services relative to GDP is a potentially powerful tool for holding governments to account in terms of these rights. This can be supplemented by a per capita target of $86 to promote universal access to primary care services in LICs. | Medium |
| McIntyre et al. | 2013 | To provide an overview of key insights from case studies in this thematic series, undertaken in seven LMICs (Costa Rica, Georgia, India, Malawi, Nigeria, Tanzania, and Thailand) at very different stages in the transition to UHC. | Costa Rica, Georgia, India, Malawi, Nigeria, Tanzania, and Thailand populations | Malawi, Nigeria, Tanzania | Qualitative; Descriptive/Exploratory case studies | Increased tax funding is particularly important if efforts are being made to extend financial protection to people outside formal-sector employment, raising questions about the value of pursuing contributory insurance schemes for this group. The prioritisation of insurance scheme coverage for civil servants in the first instance in some LMICs also raises questions about the most appropriate use of limited government funds. | High |
| McIntyre et al. | 2018 | To provide an overview of key debates and questions that have driven research around healthcare financing in SSA and key empirical findings of this research; provide an overview of how health services are financed and some of the challenges faced; and outline priority areas for future research. | Sub-Saharan African population | Sub-Saharan Africa | Mixed methods (qualitative and quantitative): Descriptive cross-sectional study | There is an urgent need for health economists to support MoHs in assessing the efficiency of use of existing government funds, identifying ways of promoting efficiency, equity, and health service quality, and compiling evidence that demonstrates strong performance in this regard. This is critical if MoHs are to succeed in making an effective case for adequate funding of health services from government revenue, without compromising allocations to other social services that are important social determinants of health. | High |
| Mulenga and Ataguba | 2017 | To assess, for the first time, the progressivity of health financing and how it impacts on income inequality in Zambia. | 19,397 households (i.e. 102,882 individuals) of the Zambian Living Conditions and Monitoring Survey | Zambia | Quantitative; analysis of health expenditure data | The extent to which taxes will be relied upon for achieving UHC in Zambia will depend inter alia on other critical issues such as the tax base, tax buoyancy and the willingness of the government to devote more resources to the health sector. | High |
| Munge et al. | 2019 | To examine the purchasing practices of micro health insurance (MHI) in Kenya. | Kenyan population | Kenya | Qualitative; Descriptive/Exploratory case studies | Specific to MHI, frameworks should support the integration of MHIs into the broader health financing system in a way that would enhance progress towards UHC. The frameworks should seek to align provider selection, performance monitoring, incentives and sanctions, so that clear and consistent signals are received by providers from all purchasing mechanisms present within the health system. | High |
| Okech and Lelegwe | 2015 | To critically review the various initiatives that the government of Kenya has over the years initiated towards the realization of UHC and how this has impacted health equity. | Government of Kenya | Kenya | Mixed methods (qualitative and quantitative): Descriptive cross-sectional study | Key issues that merit attention will include fast tracking the enactment of the Health Act currently under deliberation in parliament; increase investment in health care by considering mechanisms that embrace social solidarity; efficiency in allocation and utilization of the funds; reviewing and harmonizing the scheme of service across counties. | High |
| Okungu and McIntyre | 2019 | To critically examine the financial potential of informal sector entities, assessing their ability to prepay for health care and guide decisions regarding the most appropriate approach to financing UHC in Kenya. | Kenyan population | Two counties, Mombasa (urban) and Nyeri (rural) in Kenya | Mixed methods (qualitative and quantitative): Descriptive cross-sectional study | It is critical that Kenya, and other LMICs, pay far greater attention to understanding the nature of the informal sector within their country and designing tax systems that draw revenue from both the formal and informal sectors in the most equitable, efficient, and sustainable way to contribute to moving toward UHC. Every country, regardless of the wealth status, can increase domestic revenue for health by improving efficiency in tax collection, adjusting tax rates, and innovative financing mechanisms. | High |
| Okungu et al. | 2017 | To critically assess the financial requirements of both contributory and non-contributory mechanisms to financing UHC in Kenya in the context of large informal sector populations. | Kenyan population | Kenya | Quantitative; simulation insurance modelling | Although both funding options would require considerable government subsidies, given the magnitude of the informal sector in Kenya and their limited financial capacity, a tax-funded system would be less costly and more sustainable in the long-term than an insurance scheme approach. However, more innovative financing for health care as well as giving the health sector higher priority in government expenditure will be required to make the non-contributory financing mechanism more sustainable. | High |
| Ota et al. | 2018 | To report the conclusions of the first-ever Africa Health Forum in June 2017 with the theme “Putting People First: The Road to UHC in Africa”. The Forum aimed to strengthen and forge new partnerships, align priorities and galvanize commitment to advance the health agenda in Africa to attain UHC and the SDGs. | 800 Africa Health Forum Attendees | Africa | Qualitative; Descriptive/Exploratory; Review of forum proceedings | The deliberations culminated into a “Call-to-Action” – Putting People First: The Road to UHC in Africa, which pledged a renewed determination for Member States, in partnership with the private Sector, WHO, other UN Agencies and partners to support the attainment of the SDGs and UHC. There was agreement that immediate action was required to implement the call-to-action, and that the WHO African Regional Office should develop a plan to rapidly operationalize the outcomes of the meeting. | High |
| Pettigrew and Mathauer | 2016 | To assess voluntary health insurance (VHI) expenditure trends in LMIC and explore possible explanations. | LMICs | LMICs | Quantitative; analysis of health expenditure data | Expanding VHI markets bear the risk of increasing fragmentation and inequities. To avoid this, health financing strategies need to be clear regarding the role given to VHI on the path towards UHC. This is an important lesson for countries on the path to UHC, suggesting that although VHI may be considered to help increase pre-payment mechanisms and reduce financial risk in the first instance, if vested interests become significant this may hinder future efforts to expand government health expenditure. | Medium |
| Reeves et al. | 2015 | To investigate how alternative tax systems affect the breadth, depth, and height of health system coverage. | 89 LMICs | 89 LMICs | Quantitative; analysis of health expenditure data | Increasing domestic tax revenues is integral to achieving UHC, particularly in countries with low tax bases. While raising already high taxes further might yield less revenue, the low tax rates in LMICs suggest a wide scope to increase them. Progressive tax policies within a pro-poor framework might accelerate progress toward achieving major international health goals. | High |
| Sambo and Kirigia | 2014 | To prepare a synthesis on the situation of health systems’ components, to analyse the correlation between the interventions related to the Millennium Development Goals (MDGs) and health systems’ components and to outline four major thrusts for progress towards UHC. | 47 Member States of the World Health Organization (WHO) African Region | WHO AFRO Region** | Qualitative; Descriptive/Exploratory | We propose four thrusts for attainment of the UHC goal: strengthening public health infrastructure capacity, raising sufficient resources to strengthen health systems, promoting efficiency in national health services to optimise resource use and maximise results, and removing financial risks and barriers to care and service access. | Medium |
| Sanogo et al. | 2019 | To examine the effects of UHC in facilitating equitable access to care in Africa amongst underprivileged individuals and communities. | Ghana, Kenyan, Malawi, Madagascar, Burkina Faso and Rwandan populations | Ghana, Kenya, Malawi, Madagascar, Burkina Faso and Rwanda | Systematic review | A system to control medicines and prevent material shortages and a strong regulation of the financial system for rebate payments to health professionals is recommended. Through the increase of coverage by health insurance schemes, there can be improvements in access to care and thereby positive health outcomes in African populations. The private sector through PPPs could be involved to address quality, efficiency, and financing issues in the health infrastructure and service delivery. | High |
| Ssennyonjo et al. | 2018 | To document and analyze government resource contributions (GRCs) to private-not-for-profit (PNFP) providers in Uganda from 1997 to 2015. To understand the processes, mechanisms and dynamics of GRCs and analyze how government-PNFP relationships have adapted over time. | Primary Health Care (PHC) grants to the Uganda Catholic Medical Bureau | Uganda | Mixed methods (qualitative and quantitative): Interviews and secondary quantitative data | GRCs could be leveraged to mitigate the financial burden on communities served by PNFPs. Governments seeking to advance UHC goals should explore policies to expand GRCs and other modalities to subsidize the operational costs of PNFPs. As the agenda for UHC takes center stage, contributions of resources from government to PNFPs should be revisited; financial allocations should be increased, and strategic purchasing arrangements established that create explicit performance expectations for government funds. | High |
| Uzochukwu et al. | 2015 | To provide an overview of the state of health care financing in Nigeria, including policies in place to enhance healthcare financing. | Nigerian population | Nigeria | Systematic review | The following strategies are recommended if Nigeria is to achieve UHC: (i) replacement of OOPs with more equitable modes of financing; (ii) articulate clear policies on PHC financing; (iii) more clarity about the roles of different levels of government in financing PHC, and which components are to be financed by each level of government; (iv) governments to give higher priority to health in their budget allocations; (v) pass and implement the national health bill; and (vi) explore innovative ways of mobilizing funds and financing health. Overall, tax‑based health financing is recommended. | Medium |
| van den Heever | 2016 | To compare the recent recommendations, referred to here as National Health Insurance version 2 (NHI 2), to earlier reforms that defined the period from 1994 to 2008, named National Health Insurance version 1. | South African population | South Africa | Qualitative; Descriptive/Exploratory; health reform review | Even with the final implementation of NHI 2, there is no reason why both substitutive and supplementary coverage in the private system should not form part of the formal UHC approach - provided pooling, both vertical and horizontal, are maintained at a societal level. | Medium |
| Wang and Rosemberg | 2018 | To describe Tanzania’s efforts to promote UHC inclusive of the poor, and to identify challenges and opportunities for the health system to advance in a coherent and integrated fashion. | Tanzanian population (53 million inhabitants) | Tanzania | Qualitative; Descriptive/Exploratory case studies | The core of the reforms proposed by the Tanzania’s Health Financing Strategy (HFS) consists of the creation of a mandatory Single National Health Insurance for all Tanzanians. It will have a single risk and financial pool, which allows for cross-subsidization between the rich and the poor. The HFS sets an explicit strategic objective of establishing a pro-poor financing mechanism. | High |
| Wang et al. | 2018 | To conduct a technical analysis of public funds allocation mechanisms in Tanzania. | Tanzanian population (57 million in 2017) | Tanzania | Qualitative; Descriptive/Exploratory case studies | A health financing strategy has been developed, envisioning a single national health insurance for the entire country, guaranteed coverage of the poor, and movement toward output-based payment mechanisms. A roadmap that prioritizes poor areas in investment of key inputs will be indispensable to ensure that health human resources, operating expenses, and infrastructure upgrades are improved in an integrated manner. When transitioning to output-based financing, as the health financing strategy outlines, it is worth exploring some output measures related to how the poor are being reached and served. | Medium |

**Note: Fragile and conflict-affected settings (FCAS) include Afghanistan; Angola; Bosnia & Herzegovina; Burundi; Cameroon; Central African Republic; Cambodia; Chad; Comoros; Congo Rep; Côte d'Ivoire; Djibouti; Democratic Republic of Congo; Eritrea; Gambia, The Georgia; Guinea; Guinea-Bissau; Haiti; Iraq; Kiribati; Kosovo; Lao PDR; Lebanon; Liberia; Libya; Madagascar; Malawi; Mali; Mauritania; Marshall Islands; Micronesia; Myanmar; Nigeria; Nepal; Palestine (West Bank & Gaza); Papua New Guinea; São Tomé and Princípe; Sierra Leone; Solomon Islands; Somalia; South Sudan; Sudan; Syria; Tajikistan; Timor Leste; Togo; Tonga; Tuvalu; Uzbekistan; Vanuatu; Yemen; Zimbabwe; Pakistan; Rwanda; Uganda; and Ukraine.*

***Note: WHO African Region is composed of Algeria, Angola, Benin, Botswana, Burkina Faso, Burundi, Cabo Verde, Cameroon, Central African Republic, Chad, Comoros, Congo, Côte d'Ivoire, Democratic Republic of Congo, Equatorial Guinea, Eritrea, Eswatini, Ethiopia, Gabon, Gambia, Ghana, Guinea, Guinea Bissau, Kenya, Lesotho, Liberia, Madagascar, Malawi, Mali, Mauritania, Mauritius, Mozambique, Namibia, Niger, Nigeria, Rwanda, São Tomé and Princípe, Senegal, Seychelles, Sierra Leone, South Africa, South Sudan, Togo, Uganda, United Republic of Tanzania, Zambia and Zimbabwe.*
